# Does becoming a parent reduce sports participation? A longitudinal study of short- and long-term effects

**DOI:** 10.3389/fspor.2025.1504793

**Published:** 2025-02-13

**Authors:** Hidde Bekhuis, Femke van Abswoude

**Affiliations:** Department of Orthopedagogics: Learning, Education and Development, Behavioural Science Institute, Radboud University, Nijmegen, Netherlands

**Keywords:** parenthood, sport participation, sport behaviour, gender differences, longitudinal data

## Abstract

**Introduction:**

Parenthood can have a negative effect on sport behaviour despite the known health benefits of participation in sports. Recent studies have shown that becoming a parent is related to a reduction in exercise. However, this relationship is less clear for men than women. In addition, most studies only focused on short-term effects. Therefore, it is unknown whether these effects remain prevalent one year after becoming a parent.

**Method:**

Using twelve data waves of the Dutch Longitudinal Internet studies for the Social Sciences (LISS) panel, we examined the influence of becoming a parent on sport behaviour in the short- (<1 year) and long-term (>1 year). Given the known differences between men and women, we also examined possible gender differences in this change. Multilevel logistic regression of the data of 6,276 observations for 725 respondents showed that the short- and long-term effects of becoming a parent have different implications for the sport behaviour of men and women.

**Results:**

While men's participation in sports is not affected by parenthood, women initially stop participating in sports, but they start again after one year. Additionally, women's frequency of engagement in sports is reduced when they become mothers. In contrast, the frequency of sport engagement is not affected when men become fathers. From a resource perspective, the results show how limited time and energy can differentially affect the sport behaviour of men and women after they become parents.

**Discussion:**

Together with the different pathways of sport participation in the short- and long-term, these results can inform the development of interventions aimed at sustainable physical activity for new parents.

## 1 Background

### 1.1 Relevance and research question

Our society has reached a pinnacle in physically inactive behaviour, which affects people in all age groups and from all socioeconomic levels (1). An inactive lifestyle contributes to increased health issues, decreased participation in society, work disability, and social isolation (2–4). Sustainable improvement of the level of physical activity in society is therefore important (5). A way to study this is to focus not on why people start participating in sports but instead why they stop.

There is evidence that during major life transitions, such as the transition from primary to secondary school or into parenthood, sport participation changes (6, 7). Most, but not all, studies show that during these transitions, the frequency of sport participation decreases or that people stop playing sports (8). However, some studies suggest that the impact of life transitions is more subtle (7). People do not decrease their frequency of sport participation or stop playing sports but rather change their sport behaviour, for example, from participating in team sports to participating in individual sports.

To promote an active society, the transition into parenthood might be the most important focus. First, the evidence for the negative impact of this transition on sport behaviour is the most pronounced compared to those of other life transitions, such as the transition from primary to secondary education, getting married, starting the first job or moving (8). In addition, the sport behaviour of parents has a large influence on the sport behaviour of their children (9, 10). Finally, when children participate in sports during childhood, they are more likely to continue their sport behaviour later in life (11, 12). Therefore, understanding the short- and long-term effects of becoming a parent on sport behaviour can provide valuable insights into promoting a sustainable active society.

The majority of recent review studies (8, 13, 14) examining the influence of parenthood on physical activity have focused on nonsport activities (such as commuting or gardening). Although the main outcomes indicate that becoming a parent is related to a decrease in physical activity, this relationship is less clear for men than for women (8). In addition, most longitudinal studies have focused on the effects within the first year after childbirth (8, 13, 14). Therefore, it is unknown whether these effects remain constant after a longer period of time. In this study, we used longitudinal data up to twelve years to examine the impact of becoming a parent on sport behaviour. Using twelve waves of the representative Dutch LISS panel with 165 persons who became parents during the data collection period, we filled the abovementioned knowledge gaps by (1) examining the influence of becoming a parent on sport behaviour in the short (<1 year) and long term (>1 year) and (2) focusing on the possible gender difference in this sport behaviour. Consequently, the main question this study sought to answer was as follows: How does the sport behaviour of people who become parents change in the short and long term, and does this change differ between men and women?

### 1.2 Theory and hypotheses

#### 1.2.1 Short-term effects: limited resources

When (social) action is viewed from a resource perspective (15–17), becoming a parent reduces the number of resources for sport participation since being a parent leads to scarce time (and energy) resources (18). Alternatively, people may perceive that they have less time or energy for sports (19). Since playing a sport is a relatively informal and optional activity, the experience of time pressure leads to individuals giving up this activity when more formal and obligatory tasks require attention, such as childcare (20). This is the most likely explanation for the decrease in sport behaviour that was found in previous studies (8, 14, 21). Consequently, we expected to find a similar immediate decrease in both sport participation (Hypothesis 1a) and the frequency of sport participation (Hypothesis 1b).

In addition to this general decrease, we expected to observe gender differences. In general, women participate less in sports than men (22). Although parenting is becoming more equal between fathers and mothers, in western societies, there is still a large difference in childcare responsibilities between men and women, where women have (many) more responsibilities (23). As a result of this unequal distribution, mothers have less free time (24); hence, the time available for mothers to play sports is reduced compared to that for fathers (25). In addition, women have to give birth, which is physically demanding. Although from a gynaecological point of view, women are considered to be recovered six weeks after giving birth (26), it could take much longer to become fit enough to participate in sports again (27). Therefore, we expected that the decrease in sport participation (Hypothesis 2a) and the frequency of sport participation (Hypothesis 2b) would be more pronounced for women than for men.

Regardless of gender, the time restraints after becoming a parent do not automatically mean that people cease their sport participation altogether. They could also lead to a change in sport behaviour (7). Team sports, such as volleyball and football, are more time restricting due to fixed, and often mandatory, training times and sessions. In contrast, individual sports that can be performed at home, such as running and cycling, can be done whenever one prefers, without any restrictions or obligations (28). Although sports clubs—and especially team sports—have social value (e.g., friendships and fun) (29, 30), this often does not outweigh the time demands (7). Consequently, we expected parents to shift from team sports to more individual types of sports (Hypothesis 3).

#### 1.2.2 Long-term effects: resources and cultural barriers

While the direct impact of parenthood may reduce the time for and prioritization of sports, the decrease in sport behaviour can take place over time. That is, as children grow older, they become more independent, especially when they start attending primary school. With this, the main tasks of parents shift from caring to educating (31). In addition, parenting becomes different over time; while in the beginning of parenthood, everything is still new and exciting, time and experience could make parenting come more naturally (32, 33). This could indicate that parents gradually have more time for themselves. Thus, we expected a rebound effect indicating that parents’ sport participation (Hypothesis 4a) and frequency of sport participation (Hypothesis 4b) would increase as their children grew older.

In addition, we anticipated gender differences in this rebound effect, which should be opposite to the gender effect expected in the short term. First, because women are expected to have a larger decrease in sport participation and the frequency of sport participation in the short term, larger possibilities for the rebound effect result in the long term. Moreover, we anticipated that some of new mothers would stop participating in sports due to physical problems caused by pregnancy or giving birth (27). The majority of these physical problems would disappear or diminish over time, resulting in an increased ability to participate in sports. Consequently, we expected that the rebound effect would be more pronounced for women than for men (Hypothesis 5).

Although we believed that the resources of parents for sports would increase over time, it seemed unlikely that this would result in increased participation in team sports, given the previously mentioned demands of this type of sport (28). It could be expected that parents would still want or require the flexibility to schedule their sport activities to include them into their other daily or weekly routines. These routines often do not overlap with the specific times of practices and games for team sports. Hence, we expected that as children got older parents will switch from team sports to individual sports (Hypothesis 6).

## 2 Methods

### 2.1 Data

The present study used the Longitudinal Internet Studies for the Social Sciences (LISS) panel developed by CentERdata (Tilburg University, The Netherlands), which can be freely downloaded from https://www.lissdata.nl/. The LISS panel started in 2007 and is based on a true probability sample of households from the Netherlands drawn from the population register by Statistics Netherlands. The LISS panel involves an internet-based panel that uses incentive payments to increase the response rate. Approximately 80% of the eligible persons living in the registered panel households participate in the panel (34). The monthly response rate of these participants varies between approximately 50% and 80%, depending on the questionnaire and month.

A study of the representativeness of the LISS panel in the first year after recruitment showed that some specific groups, for example, elderly women and nonwestern first-generation immigrants, were initially somewhat underrepresented in the LISS panel (35). Another study compared the composition of the LISS panel between April 2008 and April 2010 with the Dutch population (36), showing the same underrepresentation.

The respondent attrition rate is approximately 12% per year, and the household attrition rate is 10% per year. Descriptions of which specific demographic groups have especially high or low probabilities of becoming inactive or dropping out are given by De Vos (36, 37). To address attrition, new representative samples were drawn from the Dutch population in 2009, 2011, 2013 and 2014 to have enough respondents who reflect the Dutch population regarding (1) household type, (2) age, and (3) ethnicity.

### 2.2 Respondents

For the period 2008–2019, we used the individual-level data for the head of the household from the LISS “background variables” (LISS project number 1) “social integration and leisure” (LISS project number 4), “family and household” (LISS project number 5) and “work and schooling” (LISS project number 6) modules, which were collected yearly.^1^ We only selected the head of the household since this household member completed most of the LISS questionnaires. In addition, we only selected respondents who filled out each module at least two times without any missing values for the variables described below, resulting in 725 respondents and 6,276 observations, ranging from 2 to 12 measurement points with a mean of 6.967 and an SD of 3.289.

### 2.3 Measures

#### 2.3.1 Dependent variables

To determine if participants *participated in sports*, we used the question ‘Do you practice sports?’ from the social integration and leisure module. This question could be answered as yes (1) or no (0). The mean of this question across all waves was.723, and the SD was.447.

When respondents indicated that they participated in sports, they were asked ‘How many hours do you spend playing sports per week, on average?’. This question was used to measure respondents’ *frequency of sport participation*. To be able to include nonplayers in the frequency of sport participation analyses, their scores were set to zero (0). The distribution of the number of hours spent playing sports was extremely right skewed, making these data unfit for linear regression analysis. To account for the variety in the frequency of sport participation, we recoded the number of hours spent playing sports into 4 categories: 0 h (27.69%), more than 0 and less than 2 h (12.01%), 2 or more hours and less than 4 h (31.04%) and 4 or more hours (29.25%).^2^

Respondents who indicated participating in sports were asked which sport they practiced. They could choose a maximum of three sports from a list of seventeen sports plus the option ‘other’. While the questionnaire did not include all 48 sports commonly listed in sport research guidelines, it covered the 17 most popular sports in the Netherlands. Less than 2% of respondents reported participating in sports not listed, which were excluded from the analyses. We reduced these seventeen sports to the categories *team sports* (football, handball, volleyball and indoor football; 9.38%), *individual sports at a sport centre* [badminton, squash, gymnastics, swimming, tennis, golf, fitness and music sports (aerobics, Zumba, etc.; 43.77%)], this category encompasses both sport clubs and commercial sport providers. And *individual sports at home* (running, cycling and walking; 36.31%). Because respondents could choose more than 1 sport, the percentages do not add up to 100. Our classification refines the approach by Van Houten et al. by distinguishing between team sports, individual sports at a sports center, and individual sports at home, based on resource demands such as subscription requirements and the effort needed to attend a sport location (7).

#### 2.3.2 Independent variables

After asking the question ‘Do you have children?’, which was asked in all waves, we used the question “In which year were the child(ren) born?” to determine if a respondent had *become a parent (again)* between the two waves of the survey. This occurred 165 times (2.63% of the observations) between two waves.

To measure the *number of years after having a child,* we subtracted the year of birth of the last child born from the year in which the questionnaire was administered. This means that someone who became a parent (again) between 2 waves started again at 0.^3^ In addition, for these variables, we could only include respondents who were parents, resulting in data from 449 respondents. The number of years after the birth of the last child across all waves ranged between 0 and 49 with a mean of 14.415 and an SD of 10.225.

#### 2.3.3 Control variables

We included the *age* of the respondents across all waves, which ranged from 16 to 84 years, with a mean of 44.051 and an SD of 13.870. Furthermore, we indicated if a respondent was *male* (46.69%) or *female* (53.31%) and if they had a *partner* (76.93%) or not (23.07%). Educational level was determined as the *highest completed educational level* in the year the questionnaire was administered. We included this educational level as the theoretical age at which respondents finished their education, ranging from 0 (no education at all) to 28 (PhD completed), with a mean of 19.89 and an SD of 3.034. Finally, we included the number of average *working hours* per week, ranging from 0 to 80 h, with a mean of 19.025 and an SD of 18.502.

The descriptive information of all variables can be found in Supplementary Table A1.

### 2.4 Data analysis

#### 2.4.1 Statistical methods

Our data are characterized by a multilevel structure: measures over time (years) are nested in respondents (38). Therefore, we used a two-level approach in which the yearly measures were nested within the respondents (39). In addition, we used three different dependent variables: (91) sport participation, (2) the frequency of sport participation and (3) the types of sports. The first and the last were dichotomic (0 1) variables; hence, to test our hypotheses concerning sport participation and types of sports, we used the multilevel logistic regression command “xtlogit” with robust standard errors in STATA 15.1.^4^

To test our hypotheses concerning the frequency of sport participation, which was an ordinal variable (0 h, >0 h & <2 h, ≥2 h & <4 h, ≥4 h), we performed multilevel random-effects ordered logistic models using the “xtologit” command with robust standard errors in STATA 15.1. An ordered logit uses the ranked categories of a Likert scale to create thresholds (41).^5^ The coefficients from this estimate provide the direction of change and significance but not the absolute magnitude.

#### 2.4.2 Model construction

We focused on the short-term and long-term effects of becoming a parent. Due to collinearity issues, the long-term effects could only be measured among people with children. Therefore, the short-term effect models were based on all respondents (*n* = 725) and focuses on the period of the first year of the newborn child's life. The long-term effect models were based on respondents with children (*n* = 449), and focuses on the period from when the last born child is 1 year old. The model structure was virtually the same for all types of analyses. We started with a complete model without interaction terms. For the models regarding the short-term effect, we included the variable *becoming a parent (again)* and all control variables (Model 1). In the subsequent model (Model 2), the interaction term becoming a parent * female sex was added in addition to the already included variables. For the long-term effect models, we replaced the variable *becoming a parent (again)* by *the number of years after having a child*. We added the interaction terms the number of number of years since becoming a parent * female sex.

## 3 Results

### 3.1 Sport participation in the short term

Table 1 shows the results of the multilevel logistic regression analyses on sport participation. We test our hypotheses one-tailed, because we have directed hypotheses. This means that the *p* value can be divided by 2, so we call a *p* value of 0.1 significant. Model 1 showed, in accordance with Hypothesis 1a, that becoming a parent resulted in a smaller likelihood of participating in sports (OR.678) than not becoming a parent. Moreover, the likelihood of women participating in sports was almost half that of men (OR.594). Furthermore, it appeared that age influenced sport participation, with increasing age leading to a smaller chance of participating in sports (OR.976). The other control variables did not significantly affect sport participation.

**Table 1:** Multilevel logistic regression on sport participation.

	Model 1					Model 2				
	Odds ratio	Std. Err.	95% CI		*p*	Odds ratio	Std. Err.	95% CI		*p*
Becoming parent										
Not a parent as the reference	**0** **.** **678**	0.142	0.449	1.022	0.063	1.177	0.426	0.579	2.391	0.653
Sex										
Male as the reference	**0**.**594**	0.100	0.427	0.828	0.002	**0**.**610**	0.103	0.438	0.851	0.004
Becoming a parent * female sex						**0**.**403**	0.182	0.166	0.977	0.044
Age	**0.976**	0.006	0.963	0.988	0.000	**0**.**976**	0.006	0.963	0.988	0.000
Educational level	1.016	0.024	0.970	1.065	0.507	1.016	0.024	0.969	1.065	0.510
Partner										
No partner as a reference	1.023	0.170	0.739	1.417	0.889	1.028	0.171	0.743	1.424	0.867
Working hours	1.001	0.004	0.993	1.009	0.773	1.001	0.004	0.993	1.009	0.797
Constant	14.201	9.132	4.027	50.082	0.000	13.970	9.010	3.947	49.449	0.000
Variance										
Within individuals	1.832	0.078				1.832	0.078			
Between individuals	1.211	0.085				1.211	0.085			
Log pseudolikelihood	−3,081.33					−3,079.35				

Source: LISS 2008–2019. *N* = 6,276, parents = 725.

Bold values have a significant effect *p* < 0.1.

**Figure 1:**
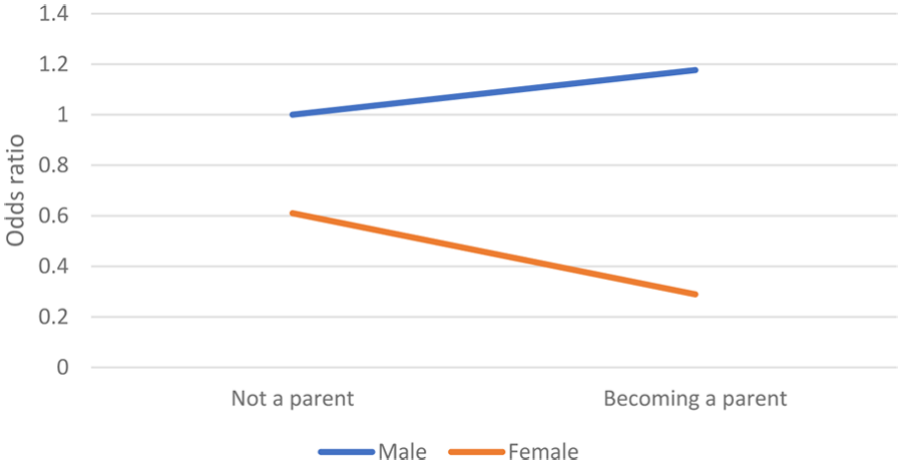
Likelihood of participating in sports.

In Model 2, the interaction term becoming a parent * female sex was added to determine the effect of becoming a parent for men and women. It appeared that, in accordance with Hypothesis 2a, the effect of becoming a parent was much smaller for men than for women. Figure 1 shows this effect graphically. Men without children were the reference group, with an odds ratio of 1. Men who became fathers had a higher likelihood of participating in sports (OR 1.177); however, this effect was not significant. The likelihood of women without children participating in sports was almost 40% lower (which is the main gender effect, b = −0.494 and OR = 610). However, the likelihood of women who became mothers was even smaller (this was the main gender effect b = −0.494 + the interaction term b = −0.910 results in a b −1.404 with an OR of 0.289). Hence, the effect of becoming a parent on sport participation was more than five times larger for women than for men.

### 3.2 Sport participation in the long term

**Figure 2:**
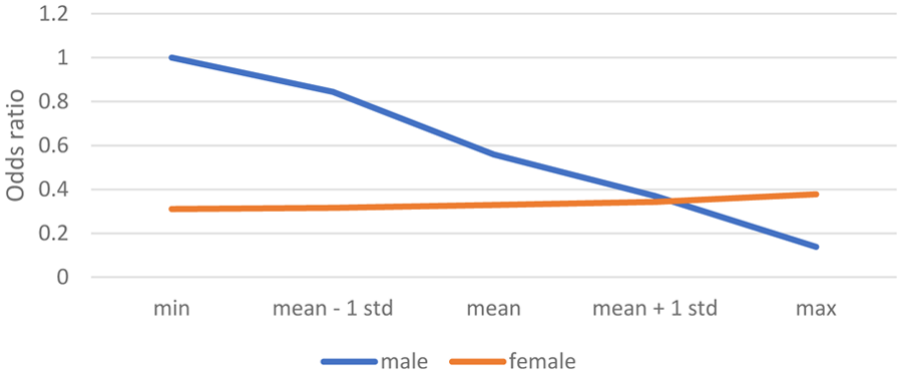
Likelihood of participating in sports according to the number of years since becoming a parent.

Supplementary Table A2 shows the results of the multilevel logistic regression analyses on sport participation with the variable the *number of years after having a child* included instead of the variable becoming a parent. It appeared that in Model 1, only women seemed to have a smaller likelihood of participating in sports than men (OR.555). In contrast to our expectation formulated in Hypothesis 4a, the number of number of years since becoming a parent did not significantly affect the likelihood of participating in sports. However, from Model 2, it appeared that the interaction variable the number of years since becoming a parent * female sex had a significant effect, which is graphically shown in Figure 2. Men with 0 years since becoming a parent were the reference category, with an OR of 1. While the likelihood of men's sport participation seemed to decrease over the years after they had become a parent, this effect was not significant. In contrast, women's likelihood of sports participation increased little, but significantly over time (the OR increased from.310 to.378). Hence, the data showed no overall rebound effect of sport participation after having a child, but they did show a rebound effect only for women, which is in accordance with Hypothesis 5.

### 3.3 Frequency of sport participation in the short term

Supplementary Table A3 shows the results of the multilevel random-effects ordered logistic models. The first model showed that, in accordance with Hypothesis 1b, becoming a parent resulted in a decreased likelihood of a higher frequency of sport participation (OR.663). In addition, and similar to the effect on sport participation, women had a much smaller likelihood than men (OR.451). Finally, the control variables indicated that with increased age, the likelihood of a high frequency of sport participation decreased, whereas the odds increased when people had a partner.

**Figure 3:**
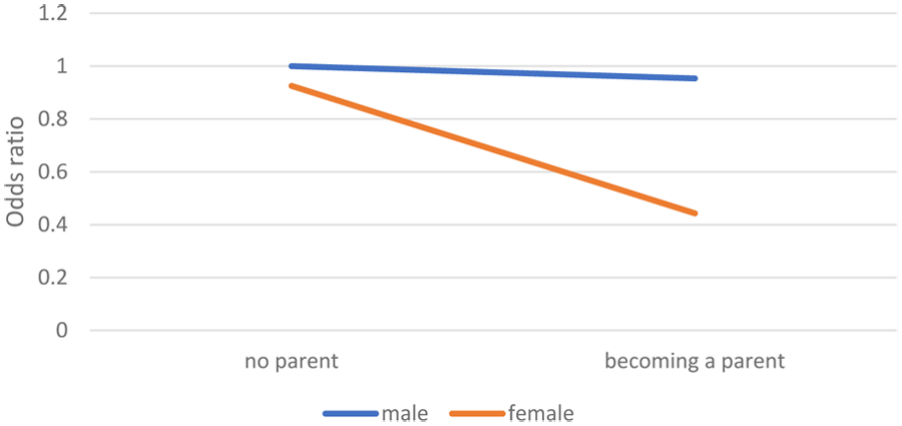
Changes in the frequency of sport participation.

In Model 2, as shown in Supplementary Table A3, we included the interaction variable becoming a parent * female sex, which is graphically represented in Figure 3. Again, men without children were the reference category, with an OR of 1. Becoming a parent had a small negative but nonsignificant effect on the likelihood of men's frequency of sport participation. For women, however, the effect of giving birth on the frequency of sport participation was significant and much larger. Women who did not have children had an OR of.925, and they participated in sports slightly less frequently in comparison to men who did not have children. Women who had children, however, had an OR of.443, which was much lower. This gender difference is in accordance with Hypothesis 2b.

### 3.4 Frequency of sport participation in the long term

Supplementary Table A4 shows the possible rebound effect of the number of years since becoming a parent on the frequency of sport participation. It appeared that there was no general rebound effect (Model 1) or gender-specific rebound effect (Model 2) for the frequency of sport participation, as predicted in Hypotheses 4b and 5. It only appeared that women had a much smaller likelihood of a high frequency of sport participation compared to men.

### 3.5 Types of sports played in the short term

Finally, we analysed the effect of becoming a parent on the types of sports played in the short term and in the long term. The models of the multilevel logistic regression analyses are shown in Supplementary Table A5. In the short term, we found that becoming a parent had a negative effect only on the likelihood of participating in sports at home (OR.549) and not on team sports or individual sports at another location. Hence, there was no evidence supporting Hypothesis 3, which predicted that team sports would become less popular and individual sports would become more popular. In addition, women also had a smaller likelihood of participation in team sports (OR.050). Having a partner significantly increases the odds of participating in team sports (OR 1.925).

### 3.6 Types of sports played in the long term

For long-term effects, when the number of years since becoming a parent was included in the analysis (Supplementary Table A6), we observed a significant effect of this variable on the likelihood of participating in team sports. The more years since the birth of a child, the lower the likelihood of participating in team sports (OR.851), which was in accordance with Hypothesis 6. However, we did not see a significant increase in participation in individual sports over time. In addition, we also observed a lower likelihood of women participating in both team sports (OR.112) and individual sports at home (OR.532). It appeared that having a partner more than doubled the likelihood of participating in sports at home (OR 2.165) than when someone had a child but no partner.

## 4 Discussion

### 4.1 Sport participation and the frequency of sport participation in the short term

In general, men participate in sports more than women (42, 43). Our results show that there is a gender difference for new parents and confirm that the transition into parenthood leads to a short-term decrease in sport participation and the frequency of sport participation only among mothers. This finding is in line with most of the previous literature showing that decreases in physical activity or sport participation and the frequency of sport participation are most pronounced for women (7, 8). Interestingly, while fatherhood does seem to result in a decrease in overall physical activity (14, 44), our results indicate that this is not caused by a decrease in sport behaviour. This finding may suggest that men still have enough resources for sports, despite the time and energy required by the newborn child, which may have several explanations. One possible explanation may be that men prioritize sports over other leisure time activities. For example, men use active transportation (walking and biking) less often than women (45). As a result, men may decrease their overall physical activity but may save time that can be spent participating in sports. Another, methodological, reason could be the use of different measuring instruments. What is considered sport and what is considered physical activity can differ between different studies. For example, In our study walking is considered as a sport. In the Netherlands walking is a fairly big sport where people go on walking tours to earn certificates and medals. It is therefore not strange to consider walking as a sport in the Dutch context. However, in other countries this could not be the case and walking could be seen as a leisure activity and not a sport. To exclude this discrepancies, future studies should simultaneously study sport and exercise behaviour. However, this does not explain the gender difference we found. This difference between men and women may also reflect the persistent cultural gender difference in childcare responsibilities (46, 47). Finally, the decrease in sport participation and the frequency of sport participation seen in women could also be related to the physical challenges that women experience after childbirth (27).

### 4.2 Sport participation and the frequency of sport participation in the long term

This study is, to our knowledge, unique because we tracked sport behaviour up to twelve years after a child is born. For women, we found a small but significant effect that they started playing sports again when their child(ren) grew older. This provides support for the arguments that the time demands of parenting may decrease over time (32, 33) and that the physical changes caused by pregnancy and childbirth that women may experience are diminished (27). This is also in line with the study of Hamilton and White (48), who describe that parenthood also offers new opportunities to become active. In contrast, the sports participation of men seems marginally affected as children grow older, which may be partially explained by the lack of a decrease in sports participation in the short term. However, even if women start (again) with sports when the children are older, they lag behind enormously compared to the sports behaviour of men. This suggests that cultural norms and values ​​where women have to take care of the children (46, 47) and/or sports is mainly for men (58) have a great influence.

Surprisingly, we did not find any long-term effect on the frequency of sport participation. Neither the expected rebound effect for the frequency of sport participation nor any other effects were found. We only found that men have a higher frequency of sport participation than women, regardless of the number of years since having a child, which is in accordance with the general finding that women participate in sports less than men (22). The lack of long-term effects of becoming a parent on the frequency of sport participation may be because we were unable to include frequency as an interval variable. Due to extremely right skewness of the frequency of sport participation, we had to include it as an ordinal variable with only 4 categories. Despite the fact that we checked for robustness with different category classifications, the use of 4 categories decreases the amount of information and sensitivity of the data, which may explain why we hardly found any influence of the independent variables. Further research should use analytical techniques that can address this right skewness, such as Bayesian regression (49).

### 4.3 Types of sports played in the short term

Interestingly, the effects of becoming a parent on the different types of sports were opposite to our expectations; that is, only individual sports at home were less often mentioned as the sport people participated in after becoming a parent. There are several explanations possible. First we argue that this effect can be largely explained by the membership structure of team sports and individual sports at another location. Most often, memberships for sport clubs run for a whole year (50), and by maintaining a membership, new parents can still identify themselves as participants in the sport while they don't sport at all. Unfortunately, we do not have data on the number of hours that were actually spent performing the sport (only the total number of hours per week); therefore, it could be possible that the number of hours spent performing the team sport decreased or stopped. Further research should therefore include questions about the actual number of hours that are spent playing sports at a club or commercial provider to see whether new parents who play sports at a club or commercial provider indeed continue playing sports or simply identify themselves as participants but show the same dropout rate as people who participate in individual sports at home.

Another explanation based on Durkheim's integration theory (51) is that team sports and sport club memberships result in more social integration (52) and therefore provide a high(er) level of social pressure to return to the sport after the birth of a child. However, research in Norway (53) shows that social integration is much less common at commercial sport clubs, from which we would expect a difference between team sports and individual sports at another location, which was not supported by our data. This emphasizes that future research should focus on collecting data that include the sports context in which people exercise and the actual number of hours spent playing the sport in that context. In addition, this research should distinguish between four categories; team sports, individual club sports, individual commercial sports and individual sports from home instead of the three categories we used.

### 4.4 Types of sports in the long term

In the long term, however, we observed a decrease in team sport participation, while the number of years since childbirth did not affect individual sport participation either at another location or at home. This suggests that the cessation of team sports may not be related to the transition into parenthood *per se* but rather to how the demands of team sports can be incorporated into the family schedule (28). In addition, team sports are more physically demanding than individual sports, for example, cycling or walking. Physical abilities tend to diminish over time, leading to more injuries or physical complaints, which can explain the general decrease in team sport participation over time (54). Although further research is needed that provides information about the actual frequency of sport participation according to sport type, it seems that the type of sports played is mostly affected by (time) resources and less affected by cultural barriers. However, we should mention the overrepresentation of men in team sports in the Netherlands (55) which is also the case in the sample of this study (12.48% of men and 6.47% of women indicated participation in team sports, chi^2^ = 26.78, *p* < .01). This differences is probably (partially) caused by gender norms and sport type (55) instead of resources. Further research should, therefore, (qualitatively) elaborate on the gender gap in team sports.

## 5 Conclusion

This study aimed to further understand the influence of becoming a parent on sport participation by examining both short- and long-term effects and by differentiating the effects for men and women. This was done by multilevel (ordered) logistic regression analyses on the data from 6,276 observations of 725 respondents in 12 waves of the Dutch LISS panel. Despite the limitations including the representativeness of these type of panels, the results highlight large differences between men and women. While men's sport participation does not seem to be affected by fatherhood, Women stop participating in sports in the short term, although in the long term, they show a modest recovery in their sport participation. Nevertheless, their activity levels remain lower compared to those of men. New parenthood does not affect the type of sports played by men and women differently. In the short term, individual sports at home became less popular, while in the long term, team sports became less popular.

These different pathways of sport participation over time provide valuable insights into the development of interventions aimed at new parents, which should focus mainly on women. The specific components for sustainable interventions to increase sport participation among (new) parents should follow from further (qualitative) research that disentangles mechanisms between resources (e.g., being physically able to participate in a sport and having the time and energy to do so) and cultural barriers (e.g., are mothers allowed to play sports instead of taking care of their children or do fathers facilitate that their partners participate in sport) that lead to the decrease in sport participation after becoming a parent. After all, remaining active through sports is not only beneficial for parental health (56) but also provides a great example for children to participate in sports at a later age (10, 57).

## Data Availability

Publicly available datasets were analyzed in this study. This data can be found here: https://www.dataarchive.lissdata.nl/study-units/view/1.

## Footnotes

^1^In addition to the unique respondent and household numbers, checks on gender, date of birth and, if applicable, birth year of children, were used to make sure that the data from the same respondent was used in all waves and modules. A syntax for merging the datasets is provided in the supplementary materials.

^2^We performed robustness checks with the numbers and classifications of the categories, resulting in the same conclusions.

^3^We performed robustness checks for the birth of the first (n=66), second (n=67), and third (n=27) child. It appeared that this did not affect the effect size. However, due to the lower numbers, we observed higher standard errors. Nevertheless, we concluded that our data did not show a different effect for the birth of the first, second or third child.

^4^The conventional multilevel logistic regression model incorporated cluster-specific random effects to account for the within-cluster correlation of subject outcomes: logit(Pr(*Y_ij_*=1))=*α*_0_+*α*_0*j*_+*α*_1_*x*_1*ij*_+⋯+*α_k_x_kij_*+*β*_1_*z*_1*j*_+⋯+*β_m_z_mj_*where *α*_0*j*_ ∼ *N*(0, *τ*^2^). The assumption was made that the random effects were independent of the model covariates (X,Z) (40).

^5^In this instance, the dependent variable y was established as an ordered categorical dependent variable, with the values of 1, 2, 3 and 4 as the ranked (in order of the increasing weekly frequency of sport participation) categories. Using k to represent the thresholds, a rank was established as *If y*^∗^<*k*_1_ *then y*=1, *If k*_1_<*y*^∗^<*k*_2_ *then y*=2 and so on until *If k*_3_<*y*^∗^ *then y*=4 and *y*^*^ was a latent variable (41). The probability of each rank outcome *j* could then be estimated as follows: Pr (*y_i_*=*j*)=Pr (*k_j_*_−1_ <β*X*_i_+*u_i_*<*k*_j_) (41). This showed that the probability of a particular outcome j being chosen was dependent on β*X*_i_ falling between the thresholds k_j_ and k_j-1_ for each individual (41).
