# Supplementary material for: Does becoming a parent reduce sports participation? A longitudinal study of short- and long-term effects
Source: Front Sports Act Living. 2025 Feb 13;7:1504793. doi: 10.3389/fspor.2025.1504793 (PMC11865059; doi:10.3389/fspor.2025.1504793)
Supplement: Supplementary file 1 [file Table1.docx]

**Appendix A**

| Table A1: Descriptive statistics | | | | | |
| --- | --- | --- | --- | --- | --- |
|  |  | % | M | SD | Range |
| # measurements | |  | 6.967 | 3.289 | 2-12 |
|  | |  |  |  |  |
| Variable | |  |  |  |  |
| Age | |  | 44.051 | 13.870 | 16-84 |
| Gender | Male | 46.69 |  |  |  |
|  | Female | 53.31 |  |  |  |
| Partner | Yes | 76.93 |  |  |  |
|  | No | 23.07 |  |  |  |
| Educational level (years) | |  | 19.89 | 3.034 | 0-28 |
| Working hours | |  | 19.025 | 18.502 | 0-80 |
|  |  |  |  |  |  |
| Sport participation | |  | .723 | .447 | 0-1 |
| Sport frequency | 0 hours | 27.69 |  |  |  |
|  | 0-2 hours | 12.01 |  |  |  |
|  | 2-4 hours | 31.04 |  |  |  |
|  | > 4 hours | 29.25 |  |  |  |
| Sport type | Team sports | 9.38 |  |  |  |
|  | Individual from accommodation | 43.77 |  |  |  |
|  | Individual from home | 36.31 |  |  |  |
| Becoming a parent | | 2.63 |  |  |  |
| Years after childbirth | |  | 14.415 | 10.225 | 0-49 |

| Table A2: Mulitlevel logistic regression on sport participation | | | | | | | | | | | | |
| --- | --- | --- | --- | --- | --- | --- | --- | --- | --- | --- | --- | --- |
|  |  | Model 1 | | | | |  | Model 2 | | | | |
|  |  | Odds ratio | Std. Err. | 95% CI | | p |  | Odds ratio | Std. Err. | 95% CI | | p |
| Years after bec. parent | | 0.987 | 0.026 | 0.938 | 1.038 | 0.606 |  | 0.960 | 0.029 | 0.905 | 1.019 | 0.181 |
| Sex | |  |  |  |  |  |  |  |  |  |  |  |
|  | male is reference | **0.555** | 0.127 | 0.354 | 0.868 | 0.010 |  | **0.310** | 0.117 | 0.148 | 0.648 | 0.002 |
| Y.A.B.P. * female | |  |  |  |  |  |  | **1.045** | 0.025 | 0.998 | 1.095 | 0.062 |
| Age | | 1.009 | 0.025 | 0.961 | 1.060 | 0.712 |  | 1.010 | 0.026 | 0.962 | 1.062 | 0.685 |
| Educational level | | 1.026 | 0.036 | 0.958 | 1.098 | 0.463 |  | 1.030 | 0.036 | 0.962 | 1.103 | 0.401 |
| Partner | |  |  |  |  |  |  |  |  |  |  |  |
|  | none is reference | 1.236 | 0.369 | 0.688 | 2.219 | 0.479 |  | 1.223 | 0.365 | 0.681 | 2.196 | 0.501 |
| Working hours | | 0.996 | 0.005 | 0.986 | 1.006 | 0.422 |  | 0.995 | 0.005 | 0.985 | 1.005 | 0.314 |
|  |  |  |  |  |  |  |  |  |  |  |  |  |
| Constant | | 2.641 | 3.305 | 0.227 | 30.687 | 0.438 |  | 3.413 | 4.259 | 0.296 | 39.398 | 0.325 |
| Variance | |  |  |  |  |  |  |  |  |  |  |  |
|  | within individuals | 1.913 | 0.109 |  |  |  |  | 1.909 | 0.109 |  |  |  |
|  | between individuals | 1.298 | 0.114 |  |  |  |  | 1.293 | 0.114 |  |  |  |
| Log pseudolikelihood | | -1868.960 |  |  |  |  |  | -1865.926 |  |  |  |  |
| Source: LISS 2008 - 2019, *N* = 3,760, *individuals* = 449 | | | | |  |  |  |  |  |  |  |  |

| Table A3: Mulitlevel ordered logistic regression on sport frequency | | | | | |  |  |  |  |  |  |  |
| --- | --- | --- | --- | --- | --- | --- | --- | --- | --- | --- | --- | --- |
|  |  | Model 1 | | | | |  | Model 2 | | | | |
|  |  | Odds ratio | Std. Err. | 95% CI | | p |  | Odds ratio | Std. Err. | 95% CI | | p |
| Becoming parent | |  |  |  |  |  |  |  |  |  |  |  |
|  | none is reference | **0.663** | 0.094 | 0.503 | 0.875 | 0.004 |  | 0.953 | 0.182 | 0.656 | 1.385 | 0.802 |
| Sex | |  |  |  |  |  |  |  |  |  |  |  |
|  | male is reference | **0.451** | 0.071 | 0.331 | 0.614 | 0.000 |  | **0.460** | 0.073 | 0.337 | 0.627 | 0.000 |
| Becoming parent * female | | |  |  |  |  |  | **0.502** | 0.141 | 0.289 | 0.871 | 0.014 |
| Age | | **0.983** | 0.006 | 0.971 | 0.995 | 0.005 |  | **0.983** | 0.006 | 0.971 | 0.995 | 0.005 |
| Educational level | | 0.973 | 0.018 | 0.938 | 1.009 | 0.134 |  | 0.973 | 0.018 | 0.938 | 1.009 | 0.136 |
| Partner | |  |  |  |  |  |  |  |  |  |  |  |
|  | none is reference | **1.486** | 0.211 | 1.125 | 1.962 | 0.005 |  | **1.491** | 0.211 | 1.129 | 1.968 | 0.005 |
| Working hours | | 0.998 | 0.003 | 0.992 | 1.004 | 0.567 |  | 0.998 | 0.003 | 0.992 | 1.004 | 0.563 |
|  |  |  |  |  |  |  |  |  |  |  |  |  |
| Cut 1 | | -2.969 | 0.508 | -3.964 | -1.974 |  |  | -2.953 | 0.509 | -3.951 | -1.956 |  |
| Cut 2 | | -2.164 | 0.506 | -3.155 | -1.174 |  |  | -2.148 | 0.507 | -3.142 | -1.154 |  |
| Cut 3 | | -0.037 | 0.505 | -1.027 | 0.954 |  |  | -0.019 | 0.507 | -1.012 | 0.974 |  |
|  |  |  |  |  |  |  |  |  |  |  |  |  |
| Variance | |  |  |  |  |  |  |  |  |  |  |  |
|  | between individuals | 3.473 | 0.272 |  |  |  |  | 3.475 | 0.273 |  |  |  |
| Log pseudolikelihood | | -7145.233 |  |  |  |  |  | -7142.991 |  |  |  |  |
| Source: LISS 2008 - 2019, *N* = 6,276, *individuals* = 725 | | | | |  |  |  |  |  |  |  |  |

| Table A4: Mulitlevel ordered logistic regression on sport frequency | | | | | |  |  |  |  |  |  |  |
| --- | --- | --- | --- | --- | --- | --- | --- | --- | --- | --- | --- | --- |
|  |  | Model 1 | | | | |  | Model 2 | | | | |
|  |  | Odds ratio | Std. Err. | 95% CI | | p |  | Odds ratio | Std. Err. | 95% CI | | p |
| Years after bec. parent | | 1.009 | 0.024 | 0.963 | 1.057 | 0.719 |  | 1.008 | 0.024 | 0.962 | 1.056 | 0.733 |
| Sex |  |  |  |  |  |  |  |  |  |  |  |  |
|  | male is reference | **0.401** | 0.082 | 0.269 | 0.598 | 0.000 |  | **0.406** | 0.083 | 0.271 | 0.606 | 0.000 |
| Y.A.B.P. * female | |  |  |  |  |  |  | 0.836 | 0.175 | 0.554 | 1.261 | 0.393 |
| Age |  | 1.013 | 0.023 | 0.969 | 1.059 | 0.573 |  | 1.012 | 0.023 | 0.968 | 1.058 | 0.589 |
| Educational level | | 1.016 | 0.027 | 0.964 | 1.069 | 0.558 |  | 1.015 | 0.027 | 0.964 | 1.069 | 0.568 |
| Partner | | 1.459 | 0.360 | 0.899 | 2.368 | 0.126 |  |  |  |  |  |  |
|  | none is reference |  |  |  |  |  |  | 1.459 | 0.360 | 0.899 | 2.367 | 0.126 |
| Working hours | | 0.996 | 0.004 | 0.989 | 1.004 | 0.335 |  | 0.996 | 0.004 | 0.989 | 1.004 | 0.339 |
|  |  |  |  |  |  |  |  |  |  |  |  |  |
| Cut 1 | | -0.669 | 0.992 | -2.613 | 1.275 |  |  | -0.706 | 0.992 | -2.651 | 1.238 |  |
| Cut 2 | | 0.233 | 0.994 | -1.715 | 2.180 |  |  | 0.196 | 0.994 | -1.752 | 2.144 |  |
| Cut 3 | | 2.579 | 0.999 | 0.620 | 4.537 |  |  | 2.542 | 1.000 | 0.583 | 4.501 |  |
|  |  |  |  |  |  |  |  |  |  |  |  |  |
| Variance | | 3.265 | 0.340 |  |  |  |  | 3.264 | 0.340 |  |  |  |
|  | between individuals |  |  |  |  |  |  |  |  |  |  |  |
| Log pseudolikelihood | | -4312.764 |  |  |  |  |  | -4312.491 |  |  |  |  |
| Source: LISS 2008 - 2019, *N* = 3,760, *individuals* = 449 | | | | |  |  |  |  |  |  |  |  |

| Table A5: Mulitlevel logistic regression on type of sport | | | | |  |  |  |  |  |  |  |  |  |  |  |  |
| --- | --- | --- | --- | --- | --- | --- | --- | --- | --- | --- | --- | --- | --- | --- | --- | --- |
|  |  | Team sport | | | | | Individual from a sports location | | | | | Individual from home | | | | |
|  |  | Odds ratio | Std. Err. | 95% CI | | p | Odds ratio | Std. Err. | 95% CI | | p | Odds ratio | Std. Err. | 95% CI |  | p |
| Becoming parent | |  |  |  |  |  |  |  |  |  |  |  |  |  |  |  |
|  | none is reference | 0.874 | 0.369 | 0.382 | 2.001 | 0.751 | 0.871 | 0.201 | 0.555 | 1.368 | 0.549 | **0.549** | 0.136 | 0.339 | 0.891 | 0.015 |
| Sex |  |  |  |  |  |  |  |  |  |  |  |  |  |  |  |  |
|  | male is reference | **0.050** | 0.029 | 0.016 | 0.156 | 0.000 | 1.346 | 0.251 | 0.933 | 1.940 | 0.112 | 0.725 | 0.138 | 0.499 | 1.054 | 0.092 |
| Age |  | **0.869** | 0.021 | 0.828 | 0.912 | 0.000 | 0.990 | 0.007 | 0.976 | 1.004 | 0.146 | 1.012 | 0.007 | 0.999 | 1.025 | 0.071 |
| Educational level | | 0.935 | 0.044 | 0.852 | 1.026 | 0.156 | 0.993 | 0.023 | 0.950 | 1.039 | 0.771 | 1.034 | 0.023 | 0.991 | 1.079 | 0.124 |
| Partner | |  |  |  |  |  |  |  |  |  |  |  |  |  |  |  |
|  | none is reference | **1.925** | 0.583 | 1.063 | 3.486 | 0.031 | 1.112 | 0.191 | 0.795 | 1.556 | 0.536 | 1.183 | 0.197 | 0.854 | 1.641 | 0.312 |
| Working hours | | 1.001 | 0.007 | 0.987 | 1.014 | 0.912 | 1.003 | 0.004 | 0.996 | 1.010 | 0.448 | 1.005 | 0.004 | 0.998 | 1.012 | 0.179 |
|  |  |  |  |  |  |  |  |  |  |  |  |  |  |  |  |  |
| Constant | | 1.000 | 1.522 | 0.051 | 19.738 | 1.000 | 0.884 | 0.527 | 0.275 | 2.841 | 0.836 | 0.107 | 0.065 | 0.033 | 0.350 | 0.000 |
| Variance | |  |  |  |  |  |  |  |  |  |  |  |  |  |  |  |
|  | within individuals | 5.690 | 0.601 |  |  |  | 2.192 | 0.105 |  |  |  | 2.202 | 0.103 |  |  |  |
|  | between individuals | 3.477 | 0.211 |  |  |  | 1.570 | 0.096 |  |  |  | 1.579 | 0.094 |  |  |  |
| Log pseudolikelihood | | -938.516 |  |  |  |  | -3363.146 |  |  |  |  | -3162.964 |  |  |  |  |
| Source: LISS 2008 - 2019, *N* = 6,276, *individuals* = 725 | | | | |  |  |  |  |  |  |  |  |  |  |  |  |

| Table A6: Mulitlevel logistic regression on type of sport | | | | |  |  |  |  |  |  |  |  |  |  |  |  |
| --- | --- | --- | --- | --- | --- | --- | --- | --- | --- | --- | --- | --- | --- | --- | --- | --- |
|  |  | Team sport | | | | | Individual from a sports location | | | | | Individual from home | | | | |
|  |  | Odds ratio | Std. Err. | 95% CI | | p | Odds ratio | Std. Err. | 95% CI | | p | Odds ratio | Std. Err. | 95% CI |  | p |
| Years after bec. parent | | **0.851** | 0.058 | 0.745 | 0.973 | 0.018 | 1.012 | 0.025 | 0.964 | 1.062 | 0.632 | 0.998 | 0.026 | 0.947 | 1.051 | 0.941 |
| Sex |  |  |  |  |  |  |  |  |  |  |  |  |  |  |  |  |
|  | male is reference | **0.112** | 0.052 | 0.045 | 0.279 | 0.000 | 1.460 | 0.359 | 0.901 | 2.364 | 0.124 | **0.532** | 0.139 | 0.319 | 0.887 | 0.016 |
| Age |  | 1.024 | 0.056 | 0.919 | 1.140 | 0.672 | 0.999 | 0.025 | 0.951 | 1.050 | 0.968 | 1.014 | 0.026 | 0.964 | 1.066 | 0.599 |
| Educational level | | 1.009 | 0.078 | 0.868 | 1.173 | 0.905 | 1.009 | 0.034 | 0.945 | 1.077 | 0.797 | 1.012 | 0.032 | 0.951 | 1.076 | 0.716 |
| Partner | |  |  |  |  |  |  |  |  |  |  |  |  |  |  |  |
|  | none is reference | 1.948 | 0.997 | 0.714 | 5.313 | 0.193 | 1.121 | 0.365 | 0.593 | 2.120 | 0.726 | **2.165** | 0.635 | 1.218 | 3.848 | 0.009 |
| Working hours | | 1.012 | 0.009 | 0.995 | 1.031 | 0.170 | 0.997 | 0.005 | 0.987 | 1.006 | 0.457 | 0.996 | 0.004 | 0.987 | 1.005 | 0.391 |
|  |  |  |  |  |  |  |  |  |  |  |  |  |  |  |  |  |
| Constant | | 0.002 | 0.005 | 0.000 | 0.406 | 0.022 | 0.364 | 0.419 | 0.038 | 3.471 | 0.380 | 0.117 | 0.134 | 0.012 | 1.111 | 0.062 |
| Variance | |  |  |  |  |  |  |  |  |  |  |  |  |  |  |  |
|  | within individuals | 5.207 | 0.603 |  |  |  | 2.169 | 0.136 |  |  |  | 2.281 | 0.140 |  |  |  |
|  | between individuals | 3.300 | 0.232 |  |  |  | 1.548 | 0.125 |  |  |  | 1.649 | 0.123 |  |  |  |
| Log pseudolikelihood | | -582.800 |  |  |  |  | -2007.879 |  |  |  |  | -1900.742 |  |  |  |  |
| Source: LISS 2008 - 2019, *N* = 3,760, *individuals* = 449 | | | | |  |  |  |  |  |  |  |  |  |  |  |  |
