# Supplementary material for: Does becoming a parent reduce sports participation? A longitudinal study of short- and long-term effects
Source: Front Sports Act Living. 2025 Feb 13;7:1504793. doi: 10.3389/fspor.2025.1504793 (PMC11865059; doi:10.3389/fspor.2025.1504793)
Supplement: Supplementary file 2 [file Table2.docx]

**************SPSS syntax merging LISS data files***************************.

***************.

***************.

***************.

*08 is wave 1.

*09 is wave 2.

*10 is wave 3.

*11 is wave 4.

*12 is wave 5.

*13 is wave 6.

*14 is wave 7.

*15 is wave 8.

*16 is wave 9.

*17 is wave 10.

*18 is wave 11.

*19 is wave 12.

* Encoding: UTF-8.

*compute wave6=1.

*sort cases by nomem_encr.

get file='C:\Users\U046129\Dropbox\NWA TRAIL WP3\LISS DATA\Wave 1 gezin.sav'.

MATCH FILES /FILE=*

/FILE='C:\Users\U046129\Dropbox\NWA TRAIL WP3\LISS DATA\Wave 2 gezin.sav'

/BY nomem_encr nohouse_encr.

EXECUTE.

MATCH FILES /FILE=*

/FILE='C:\Users\U046129\Dropbox\NWA TRAIL WP3\LISS DATA\Wave 3 gezin.sav'

/BY nomem_encr.

EXECUTE.

MATCH FILES /FILE=*

/FILE='C:\Users\U046129\Dropbox\NWA TRAIL WP3\LISS DATA\Wave 4 gezin.sav'

/BY nomem_encr.

EXECUTE.

MATCH FILES /FILE=*

/FILE='C:\Users\U046129\Dropbox\NWA TRAIL WP3\LISS DATA\Wave 5 gezin.sav'

/BY nomem_encr.

EXECUTE.

MATCH FILES /FILE=*

/FILE='C:\Users\U046129\Dropbox\NWA TRAIL WP3\LISS DATA\Wave 6 gezin.sav'

/BY nomem_encr.

EXECUTE.

MATCH FILES /FILE=*

/FILE='C:\Users\U046129\Dropbox\NWA TRAIL WP3\LISS DATA\Wave 7 gezin.sav'

/BY nomem_encr.

EXECUTE.

MATCH FILES /FILE=*

/FILE='C:\Users\U046129\Dropbox\NWA TRAIL WP3\LISS DATA\Wave 8 gezin.sav'

/BY nomem_encr.

EXECUTE.

MATCH FILES /FILE=*

/FILE='C:\Users\U046129\Dropbox\NWA TRAIL WP3\LISS DATA\Wave 9 gezin.sav'

/BY nomem_encr.

EXECUTE.

MATCH FILES /FILE=*

/FILE='C:\Users\U046129\Dropbox\NWA TRAIL WP3\LISS DATA\Wave 10 gezin.sav'

/BY nomem_encr.

EXECUTE.

MATCH FILES /FILE=*

/FILE='C:\Users\U046129\Dropbox\NWA TRAIL WP3\LISS DATA\Wave 11 gezin.sav'

/BY nomem_encr.

EXECUTE.

MATCH FILES /FILE=*

/FILE='C:\Users\U046129\Dropbox\NWA TRAIL WP3\LISS DATA\Wave 12 gezin.sav'

/BY nomem_encr.

EXECUTE.

MATCH FILES /FILE=*

/FILE='C:\Users\U046129\Dropbox\NWA TRAIL WP3\LISS DATA\Wave 1 sport.sav'

/BY nomem_encr.

EXECUTE.

MATCH FILES /FILE=*

/FILE='C:\Users\U046129\Dropbox\NWA TRAIL WP3\LISS DATA\Wave 2 sport.sav'

/BY nomem_encr.

EXECUTE.

MATCH FILES /FILE=*

/FILE='C:\Users\U046129\Dropbox\NWA TRAIL WP3\LISS DATA\Wave 3 sport.sav'

/BY nomem_encr.

EXECUTE.

MATCH FILES /FILE=*

/FILE='C:\Users\U046129\Dropbox\NWA TRAIL WP3\LISS DATA\Wave 4 sport.sav'

/BY nomem_encr.

EXECUTE.

MATCH FILES /FILE=*

/FILE='C:\Users\U046129\Dropbox\NWA TRAIL WP3\LISS DATA\Wave 5 sport.sav'

/BY nomem_encr.

EXECUTE.

MATCH FILES /FILE=*

/FILE='C:\Users\U046129\Dropbox\NWA TRAIL WP3\LISS DATA\Wave 6 sport.sav'

/BY nomem_encr.

EXECUTE.

MATCH FILES /FILE=*

/FILE='C:\Users\U046129\Dropbox\NWA TRAIL WP3\LISS DATA\Wave 7 sport.sav'

/BY nomem_encr.

EXECUTE.

MATCH FILES /FILE=*

/FILE='C:\Users\U046129\Dropbox\NWA TRAIL WP3\LISS DATA\Wave 8 sport.sav'

/BY nomem_encr.

EXECUTE.

MATCH FILES /FILE=*

/FILE='C:\Users\U046129\Dropbox\NWA TRAIL WP3\LISS DATA\Wave 9 sport.sav'

/BY nomem_encr.

EXECUTE.

MATCH FILES /FILE=*

/FILE='C:\Users\U046129\Dropbox\NWA TRAIL WP3\LISS DATA\Wave 10 sport.sav'

/BY nomem_encr.

EXECUTE.

MATCH FILES /FILE=*

/FILE='C:\Users\U046129\Dropbox\NWA TRAIL WP3\LISS DATA\Wave 11 sport.sav'

/BY nomem_encr.

EXECUTE.

MATCH FILES /FILE=*

/FILE='C:\Users\U046129\Dropbox\NWA TRAIL WP3\LISS DATA\Wave 12 sport.sav'

/BY nomem_encr.

EXECUTE.

MATCH FILES /FILE=*

/FILE='C:\Users\U046129\Dropbox\NWA TRAIL WP3\LISS DATA\Wave 1 werk.sav'

/BY nomem_encr.

EXECUTE.

MATCH FILES /FILE=*

/FILE='C:\Users\U046129\Dropbox\NWA TRAIL WP3\LISS DATA\Wave 2 werk.sav'

/BY nomem_encr.

EXECUTE.

MATCH FILES /FILE=*

/FILE='C:\Users\U046129\Dropbox\NWA TRAIL WP3\LISS DATA\Wave 3 werk.sav'

/BY nomem_encr.

EXECUTE.

MATCH FILES /FILE=*

/FILE='C:\Users\U046129\Dropbox\NWA TRAIL WP3\LISS DATA\Wave 4 werk.sav'

/BY nomem_encr.

EXECUTE.

MATCH FILES /FILE=*

/FILE='C:\Users\U046129\Dropbox\NWA TRAIL WP3\LISS DATA\Wave 5 werk.sav'

/BY nomem_encr.

EXECUTE.

MATCH FILES /FILE=*

/FILE='C:\Users\U046129\Dropbox\NWA TRAIL WP3\LISS DATA\Wave 6 werk.sav'

/BY nomem_encr.

EXECUTE.

MATCH FILES /FILE=*

/FILE='C:\Users\U046129\Dropbox\NWA TRAIL WP3\LISS DATA\Wave 7 werk.sav'

/BY nomem_encr.

EXECUTE.

MATCH FILES /FILE=*

/FILE='C:\Users\U046129\Dropbox\NWA TRAIL WP3\LISS DATA\Wave 8 werk.sav'

/BY nomem_encr.

EXECUTE.

MATCH FILES /FILE=*

/FILE='C:\Users\U046129\Dropbox\NWA TRAIL WP3\LISS DATA\Wave 9 werk.sav'

/BY nomem_encr.

EXECUTE.

MATCH FILES /FILE=*

/FILE='C:\Users\U046129\Dropbox\NWA TRAIL WP3\LISS DATA\Wave 10 werk.sav'

/BY nomem_encr.

EXECUTE.

MATCH FILES /FILE=*

/FILE='C:\Users\U046129\Dropbox\NWA TRAIL WP3\LISS DATA\Wave 11 werk.sav'

/BY nomem_encr.

EXECUTE.

MATCH FILES /FILE=*

/FILE='C:\Users\U046129\Dropbox\NWA TRAIL WP3\LISS DATA\Wave 12 werk.sav'

/BY nomem_encr.

EXECUTE.

MATCH FILES /FILE=*

/FILE='C:\Users\U046129\Dropbox\NWA TRAIL WP3\LISS DATA\avars_201911_EN_1.0p.sav'

/BY nomem_encr.

EXECUTE.

*fre nomem_encr.

select if ( nomem_encr ne 809063 & nohouse_encr ne 532656).

select if ( nomem_encr ne 813662 & nohouse_encr ne 564642).

select if ( nomem_encr ne 813796 & nohouse_encr ne 570004).

select if ( nomem_encr ne 817172 & nohouse_encr ne 529110).

select if ( nomem_encr ne 817987 & nohouse_encr ne 532931).

select if ( nomem_encr ne 822189 & nohouse_encr ne 599032).

select if ( nomem_encr ne 822714 & nohouse_encr ne 521466).

select if ( nomem_encr ne 824432 & nohouse_encr ne 581030).

select if ( nomem_encr ne 826269 & nohouse_encr ne 522354).

select if ( nomem_encr ne 828118 & nohouse_encr ne 536776).

select if ( nomem_encr ne 833847 & nohouse_encr ne 535260).

select if ( nomem_encr ne 834738 & nohouse_encr ne 577587).

select if ( nomem_encr ne 836380 & nohouse_encr ne 525299).

select if ( nomem_encr ne 838327 & nohouse_encr ne 544317).

select if ( nomem_encr ne 838543 & nohouse_encr ne 579391).

select if ( nomem_encr ne 843475 & nohouse_encr ne 564224).

select if ( nomem_encr ne 844671 & nohouse_encr ne 515359).

select if ( nomem_encr ne 851187 & nohouse_encr ne 578610).

select if ( nomem_encr ne 852603 & nohouse_encr ne 548422).

select if ( nomem_encr ne 854019 & nohouse_encr ne 582635).

select if ( nomem_encr ne 859194 & nohouse_encr ne 512933).

select if ( nomem_encr ne 860034 & nohouse_encr ne 595571).

select if ( nomem_encr ne 860287 & nohouse_encr ne 520001).

select if ( nomem_encr ne 861648 & nohouse_encr ne 597671).

select if ( nomem_encr ne 862213 & nohouse_encr ne 526095).

select if ( nomem_encr ne 863888 & nohouse_encr ne 532931).

select if ( nomem_encr ne 864102 & nohouse_encr ne 584027).

select if ( nomem_encr ne 871240 & nohouse_encr ne 589410).

select if ( nomem_encr ne 884729 & nohouse_encr ne 551745).

select if ( nomem_encr ne 893319 & nohouse_encr ne 506103).

select if ( nomem_encr ne 895645 & nohouse_encr ne 583471).

select if ( nomem_encr ne 896047 & nohouse_encr ne 516802).

exe.

*fre nomem_encr.

*desc cs08a105 cs09b105 cs10c105 cs11d105 cs12e105 cs13f105 cs14g105 cs15h105 cs16i105 cs17j105.

*des cf08a037 cf09b037 cf10c037 cf11d037 cf12e037 cf13f037 cf14g037 cf15h456 cf16i456 cf17j456 cf18k456 cf19l456.

*des cf08a038 cf09b038 cf10c038 cf11d038 cf12e038 cf13f038 cf14g038 cf15h457 cf16i457 cf17j457 cf18k457 cf19l457.

*des cf08a039 cf09b039 cf10c039 cf11d039 cf12e039 cf13f039 cf14g039 cf15h458 cf16i458 cf17j458 cf18k458 cf19l458.

*des cf08a040 cf09b040 cf10c040 cf11d040 cf12e040 cf13f040 cf14g040 cf15h459 cf16i459 cf17j459 cf18k459 cf19l459.

*des cf08a041 cf09b041 cf10c041 cf11d041 cf12e041 cf13f041 cf14g041 cf15h460 cf16i460 cf17j460 cf18k460 cf19l460.

compute checkkind1 = mean (cf08a037, cf09b037, cf10c037, cf11d037, cf12e037, cf13f037, cf14g037, cf15h456, cf16i456, cf17j456, cf18k456, cf19l456).

compute checkkind2 = mean (cf08a038, cf09b038, cf10c038, cf11d038, cf12e038, cf13f038, cf14g038, cf15h457, cf16i457, cf17j457, cf18k457, cf19l457).

compute checkkind3 = mean (cf08a039, cf09b039, cf10c039, cf11d039, cf12e039, cf13f039, cf14g039, cf15h458, cf16i458, cf17j458, cf18k458, cf19l458).

compute checkkind4 = mean (cf08a040, cf09b040, cf10c040, cf11d040, cf12e040, cf13f040, cf14g040, cf15h459, cf16i459, cf17j459, cf18k459, cf19l459).

compute checkkind5 = mean (cf08a041, cf09b041, cf10c041, cf11d041, cf12e041, cf13f041, cf14g041, cf15h460, cf16i460, cf17j460, cf18k460, cf19l460).

*fre checkkind1 to checkkind5.

select if (missing(checkkind1)) or (checkkind1 = 1970) or (checkkind1 = 1976) or (checkkind1 = 1978) or (checkkind1 = 1984) or (checkkind1 = 1987) or (checkkind1 = 1988) or (checkkind1 = 1990) or (checkkind1 = 1991) or

(checkkind1 = 1992) or (checkkind1 = 1993) or

(checkkind1 = 1994) or (checkkind1 = 1995) or (checkkind1 = 1996) or (checkkind1 = 1997) or (checkkind1 = 1998) or (checkkind1 = 1999) or (checkkind1 = 2000) or (checkkind1 = 2001) or (checkkind1 = 2002) or (checkkind1 = 2003) or

(checkkind1 = 2004) or (checkkind1 = 2005) or (checkkind1 = 2006) or (checkkind1 = 2007) or (checkkind1 = 2008) or (checkkind1 = 2009) or (checkkind1 = 2010) or (checkkind1 = 2011) or (checkkind1 = 2012) or (checkkind1 = 2013) or

(checkkind1 = 2014) or (checkkind1 = 2015) or (checkkind1 = 2016) or (checkkind1 = 2017) or (checkkind1 = 2018) or (checkkind1 = 2019).

select if (missing(checkkind2)) or (checkkind2 = 1970) or (checkkind2 = 1976) or (checkkind2 = 1978) or (checkkind2 = 1979) or (checkkind2 = 1980) or (checkkind2 = 1984) or (checkkind2 = 1985) or

(checkkind2 = 1987) or (checkkind2 = 1988) or (checkkind2 = 1990)

or (checkkind2 = 1991) or (checkkind2 = 1992) or (checkkind2 = 1993) or

(checkkind2 = 1994) or (checkkind2 = 1995) or (checkkind2 = 1996) or (checkkind2 = 1997) or (checkkind2 = 1998) or (checkkind2 = 1999) or (checkkind2 = 2000) or (checkkind2 = 2001) or (checkkind2 = 2002) or (checkkind2 = 2003) or

(checkkind2 = 2004) or (checkkind2 = 2005) or (checkkind2 = 2006) or (checkkind2 = 2007) or (checkkind2 = 2008) or (checkkind2 = 2009) or (checkkind2 = 2010) or (checkkind2 = 2011) or (checkkind2 = 2012) or (checkkind2 = 2013) or

(checkkind2 = 2014) or (checkkind2 = 2015) or (checkkind2 = 2016) or (checkkind2 = 2017) or (checkkind2 = 2018) or (checkkind2 = 2019).

select if (missing(checkkind3)) or (checkkind3 = 1970) or (checkkind3 = 1976) or (checkkind3 = 1978) or (checkkind3 = 1979) or (checkkind3 = 1980) or (checkkind3 = 1984) or (checkkind3 = 1985) or

(checkkind3 = 1987) or (checkkind3 = 1988) or (checkkind3 = 1990)

or (checkkind3 = 1991) or (checkkind3 = 1992) or (checkkind3 = 1993) or

(checkkind3 = 1994) or (checkkind3 = 1995) or (checkkind3 = 1996) or (checkkind3 = 1997) or (checkkind3 = 1998) or (checkkind3 = 1999) or (checkkind3 = 2000) or (checkkind3 = 2001) or (checkkind3 = 2002) or (checkkind3 = 2003) or

(checkkind3 = 2004) or (checkkind3 = 2005) or (checkkind3 = 2006) or (checkkind3 = 2007) or (checkkind3 = 2008) or (checkkind3 = 2009) or (checkkind3 = 2010) or (checkkind3 = 2011) or (checkkind3 = 2012) or (checkkind3 = 2013) or

(checkkind3 = 2014) or (checkkind3 = 2015) or (checkkind3 = 2016) or (checkkind3 = 2017) or (checkkind3 = 2018) or (checkkind3 = 2019).

select if (missing(checkkind4)) or (checkkind4 = 1970) or (checkkind4 = 1976) or (checkkind4 = 1978) or (checkkind4 = 1979) or (checkkind4 = 1980) or (checkkind4 = 1984) or (checkkind4 = 1985) or (checkkind4 = 1987) or

(checkkind4 = 1988) or (checkkind4 = 1990)

or (checkkind4 = 1991) or (checkkind4 = 1992) or (checkkind4 = 1993) or

(checkkind4 = 1994) or (checkkind4 = 1995) or (checkkind4 = 1996) or (checkkind4 = 1997) or (checkkind4 = 1998) or (checkkind4 = 1999) or (checkkind4 = 2000) or (checkkind4 = 2001) or (checkkind4 = 2002) or (checkkind4 = 2003) or

(checkkind4 = 2004) or (checkkind4 = 2005) or (checkkind4 = 2006) or (checkkind4 = 2007) or (checkkind4 = 2008) or (checkkind4 = 2009) or (checkkind4 = 2010) or (checkkind4 = 2011) or (checkkind4 = 2012) or (checkkind4 = 2013) or

(checkkind4 = 2014) or (checkkind4 = 2015) or (checkkind4 = 2016) or (checkkind4 = 2017) or (checkkind4 = 2018) or (checkkind4 = 2019).

select if (missing(checkkind5)) or (checkkind5 = 1970) or (checkkind5 = 1976) or (checkkind5 = 1978) or (checkkind5 = 1979) or (checkkind5 = 1980) or (checkkind5 = 1984) or (checkkind5 = 1985) or (checkkind5 = 1987) or

(checkkind5 = 1988) or (checkkind5 = 1990)

or (checkkind5 = 1991) or (checkkind5 = 1992) or (checkkind5 = 1993) or

(checkkind5 = 1994) or (checkkind5 = 1995) or (checkkind5 = 1996) or (checkkind5 = 1997) or (checkkind5 = 1998) or (checkkind5 = 1999) or (checkkind5 = 2000) or (checkkind5 = 2001) or (checkkind5 = 2002) or (checkkind5 = 2003) or

(checkkind5 = 2004) or (checkkind5 = 2005) or (checkkind5 = 2006) or (checkkind5 = 2007) or (checkkind5 = 2008) or (checkkind5 = 2009) or (checkkind5 = 2010) or (checkkind5 = 2011) or (checkkind5 = 2012) or (checkkind5 = 2013) or

(checkkind5 = 2014) or (checkkind5 = 2015) or (checkkind5 = 2016) or (checkkind5 = 2017) or (checkkind5 = 2018) or (checkkind5 = 2019).

*fre checkkind1 to checkkind5.

compute ouder = 0.

if not (sysmis (checkkind1)) ouder =1.

*fre ouder.

*compute oudergewordenafgelopenjaar = 0.

*if (cf08a037 = 2007) or (cf08a037 = 2008) oudergewordenafgelopenjaar =1.

*if (cf09b037 = 2008) or (cf09b037 = 2009) oudergewordenafgelopenjaar =1.

*if (cf10c037 = 2009) or (cf10c037 = 2010) oudergewordenafgelopenjaar =1.

*if (cf11d037 = 2010) or (cf11d037 = 2011) oudergewordenafgelopenjaar =1.

*if (cf12e037 = 2011) or (cf12e037 = 2012) oudergewordenafgelopenjaar =1.

*if (cf13f037 = 2012) or (cf13f037 = 2013) oudergewordenafgelopenjaar =1.

*if (cf14g037 = 2013) or (cf14g037 = 2014) oudergewordenafgelopenjaar =1.

*if (cf15h456 =2014 ) or (cf15h456 = 2015) oudergewordenafgelopenjaar =1.

*if (cf16i456 = 2015) or (cf16i456 = 2016) oudergewordenafgelopenjaar =1.

*if (cf17j456 = 2016) or (cf17j456 = 2017) oudergewordenafgelopenjaar =1.

*fre oudergewordenafgelopenjaar.

*fre cf08a037.

*fre cf09b037.

*compute oudergewordenafgelopenjaar2 = 0.

*if (cf08a037 = 2007) or (cf08a037 = 2008) oudergewordenafgelopenjaar2 =1.

*if ((cf09b037 = 2008) or (cf09b037 = 2009)) and missing(cf08a037) oudergewordenafgelopenjaar2 =1.

*if ((cf10c037 = 2009) or (cf10c037 = 2010)) and missing(cf09b037) oudergewordenafgelopenjaar2 =1.

*if ((cf10c037 = 2010) or (cf11d037 = 2011)) and missing(cf10c037) oudergewordenafgelopenjaar2 =1.

*if ((cf12e037 = 2011) or (cf12e037 = 2012)) and missing(cf11d037) oudergewordenafgelopenjaar2 =1.

*if ((cf13f037 = 2012) or (cf13f037 = 2013)) and missing(cf12e037) oudergewordenafgelopenjaar2 =1.

*if ((cf14g037 = 2013) or (cf14g037 = 2014)) and missing(cf13f037) oudergewordenafgelopenjaar2 =1.

*if ((cf15h456 =2014 ) or (cf15h456 = 2015)) and missing(cf14g037) oudergewordenafgelopenjaar2 =1.

*if ((cf16i456 = 2015) or (cf16i456 = 2016)) and missing(cf15h456) oudergewordenafgelopenjaar2 =1.

*if ((cf17j456 = 2016) or (cf17j456 = 2017)) and missing(cf16i456) oudergewordenafgelopenjaar2 =1.

*fre oudergewordenafgelopenjaar2.

*use all.

*cross oudergewordenafgelopenjaar by oudergewordenafgelopenjaar2.

*if not missing (cf08a037) age1kid = cf08a037 - gebjaar.

*if not missing (cf09b037) age1kid = cf09b037 - gebjaar.

*if not missing (cf10c037) age1kid = cf10c037 - gebjaar.

*if not missing (cf11d037) age1kid = cf11d037 - gebjaar.

*if not missing (cf12e037) age1kid = cf12e037 - gebjaar.

*if not missing (cf13f037) age1kid = cf13f037 - gebjaar.

*if not missing (cf14g037) age1kid = cf14g037 - gebjaar.

*if not missing (cf15h456) age1kid = cf15h456 - gebjaar.

*if not missing (cf16i456) age1kid = cf16i456 - gebjaar.

*if not missing (cf17j456) age1kid = cf17j456 - gebjaar.

*fre age1kid.

*cross cf08a037 by cf17j456.

*TEMPORARY.

*select if (oudergewordenafgelopenjaar2 =1).

*fre gebjaar.

*TEMPORARY.

*select if (oudergewordenafgelopenjaar2 =1).

*fre age1kid.

*CTABLES

/VLABELS VARIABLES=burgstat oudergewordenafgelopenjaar DISPLAY=LABEL

/TABLE burgstat BY oudergewordenafgelopenjaar [COUNT F40.0]

/CATEGORIES VARIABLES=burgstat ORDER=A KEY=VALUE EMPTY=INCLUDE

/CATEGORIES VARIABLES=oudergewordenafgelopenjaar ORDER=A KEY=VALUE EMPTY=EXCLUDE

/CRITERIA CILEVEL=95.

*fre cs08a104 cs08a105.

*fre cs09b104 cs09b105.

*fre cs10c104 cs10c105.

*fre cs11d104 cs11d105.

*fre cs12e104 cs12e105.

*fre cs13f104 cs13f105.

*fre cs14g104 cs14g105.

*fre cs15h104 cs15h105.

*fre cs16i104 cs16i105.

*fre cs17j104 cs17j105.

*compute sportd = cs08a105 - cs17j105.

*fre sportd.

fre cf08a003 cf09b003 cf10c003 cf11d003 cf12e003 cf13f003 cf14g003 cf15h003 cf16i003 cf17j003 cf18k003 cf19l003 geslacht.

compute checkgeslacht = mean(cf08a003, cf09b003, cf10c003, cf11d003, cf12e003, cf13f003, cf14g003, cf15h003, cf16i003, cf17j003, cf18k003, cf19l003).

fre checkgeslacht.

select if (checkgeslacht =1 or checkgeslacht =2).

fre checkgeslacht.

recode checkgeslacht (1=0) (2=1) into female.

fre female.

compute gebj1 = 2008-cf08a004.

compute gebj2 = 2009-cf09b004.

compute gebj3 = 2010-cf10c004.

compute gebj4 = 2011-cf11d004.

compute gebj5 = 2012-cf12e004.

compute gebj6 = 2013-cf13f004.

compute gebj7 = 2014-cf14g004.

compute gebj8 = 2015-cf15h004.

compute gebj9 = 2016-cf16i004.

compute gebj10 = 2017-cf17j004.

compute gebj11 = 2018-cf18k004.

compute gebj12 = 2019-cf19l004.

compute checkgebjaar = mean (gebj1, gebj2, gebj3, gebj4, gebj5, gebj6, gebj7, gebj8, gebj9, gebj10, gebj11, gebj12).

fre checkgebjaar.

select if (checkgebjaar =1914 or checkgebjaar = 1915 or checkgebjaar = 1916 or checkgebjaar = 1917 or checkgebjaar = 1918 or checkgebjaar = 1919 or checkgebjaar = 1920 or checkgebjaar = 1921 or checkgebjaar = 1922 or checkgebjaar = 1923

or checkgebjaar = 1924 or checkgebjaar = 1925 or checkgebjaar = 1926 or checkgebjaar = 1927 or checkgebjaar = 1928 or checkgebjaar = 1929 or checkgebjaar = 1930 or checkgebjaar = 1931 or checkgebjaar = 1932

or checkgebjaar = 1933 or checkgebjaar = 1934 or checkgebjaar = 1935 or checkgebjaar = 1936 or checkgebjaar = 1937 or checkgebjaar = 1938 or checkgebjaar = 1939 or checkgebjaar = 1940 or checkgebjaar = 1941

or checkgebjaar = 1942 or checkgebjaar = 1943 or checkgebjaar = 1944 or checkgebjaar = 1944 or checkgebjaar = 1945 or checkgebjaar = 1946 or checkgebjaar = 1947 or checkgebjaar = 1948 or checkgebjaar = 1949

or checkgebjaar = 1950 or checkgebjaar = 1951 or checkgebjaar = 1952 or checkgebjaar = 1953 or checkgebjaar = 1954 or checkgebjaar = 1955 or checkgebjaar = 1956 or checkgebjaar = 1957 or checkgebjaar = 1958

or checkgebjaar = 1959 or checkgebjaar = 1960 or checkgebjaar = 1961 or checkgebjaar = 1962 or checkgebjaar = 1963 or checkgebjaar = 1964 or checkgebjaar = 1965 or checkgebjaar = 1966 or checkgebjaar = 1967

or checkgebjaar = 1968 or checkgebjaar = 1969 or checkgebjaar = 1970 or checkgebjaar = 1971 or checkgebjaar = 1972 or checkgebjaar = 1973 or checkgebjaar = 1974 or checkgebjaar = 1975 or checkgebjaar = 1976

or checkgebjaar = 1977 or checkgebjaar = 1978 or checkgebjaar = 1979 or checkgebjaar = 1980 or checkgebjaar = 1981 or checkgebjaar = 1982 or checkgebjaar = 1983 or checkgebjaar = 1984 or checkgebjaar = 1985 or checkgebjaar = 1986

or checkgebjaar = 1987 or checkgebjaar = 1988 or checkgebjaar = 1989 or checkgebjaar = 1990 or checkgebjaar = 1991 or checkgebjaar = 1992 or checkgebjaar = 1993 or checkgebjaar = 1994 or checkgebjaar = 1995 or checkgebjaar = 1996

or checkgebjaar = 1997 or checkgebjaar = 1998 or checkgebjaar = 1999 or checkgebjaar = 2000 or checkgebjaar = 2001 or checkgebjaar = 2002 or checkgebjaar = 2003).

fre checkgebjaar.

*select if (checkgebjaar>1972).

*fre oudergewordenafgelopenjaar oudergewordenafgelopenjaar2.

*fre cs08a106 cs08a107 cs08a108 cs08a109 cs08a110 cs08a111 cs08a112 cs08a113 cs08a114 cs08a115 cs08a116

cs08a117 cs08a118 cs08a119 cs08a120 cs08a121 cs08a122 cs08a123.

*fre cs09b106 cs09b107 cs09b108 cs09b109 cs09b110 cs09b111 cs09b112 cs09b113 cs09b114 cs09b115 cs09b116

cs09b117 cs09b118 cs09b119 cs09b120 cs09b121 cs09b122 cs09b123.

*fre cs10c106 cs10c107 cs10c108 cs10c109 cs10c110 cs10c111 cs10c112 cs10c113 cs10c114 cs10c115 cs10c116

cs10c117 cs10c118 cs10c119 cs10c120 cs10c121 cs10c122 cs10c123.

*fre cs11d106 cs11d107 cs11d108 cs11d109 cs11d110 cs11d111 cs11d112 cs11d113 cs11d114 cs11d115 cs11d116

cs11d117 cs11d118 cs11d119 cs11d120 cs11d121 cs11d409 cs11d122 cs11d123.

*fre cs12e106 cs12e107 cs12e108 cs12e109 cs12e110 cs12e111 cs12e112 cs12e113 cs12e114 cs12e115 cs12e116

cs12e117 cs12e118 cs12e119 cs12e120 cs12e121 cs12e409 cs12e122 cs12e123.

*fre cs13f106 cs13f107 cs13f108 cs13f109 cs13f110 cs13f111 cs13f112 cs13f113 cs13f114 cs13f115 cs13f116

cs13f117 cs13f118 cs13f119 cs13f120 cs13f121 cs13f409 cs13f122 cs13f123.

*fre cs14g106 cs14g107 cs14g108 cs14g109 cs14g110 cs14g111 cs14g112 cs14g113 cs14g114 cs14g115 cs14g116

cs14g117 cs14g118 cs14g119 cs14g120 cs14g121 cs14g409 cs14g122 cs14g123.

*fre cs15h106 cs15h107 cs15h108 cs15h109 cs15h110 cs15h111 cs15h112 cs15h113 cs15h114 cs15h115 cs15h116

cs15h117 cs15h118 cs15h119 cs15h120 cs15h121 cs15h409 cs15h122 cs15h123.

*fre cs16i106 cs16i107 cs16i108 cs16i109 cs16i110 cs16i111 cs16i112 cs16i113 cs16i114 cs16i115 cs16i116

cs16i117 cs16i118 cs16i119 cs16i120 cs16i121 cs16i409 cs16i122 cs16i123.

*fre cs17j106 cs17j107 cs17j108 cs17j109 cs17j110 cs17j111 cs17j112 cs17j113 cs17j114 cs17j115 cs17j116

cs17j117 cs17j118 cs17j119 cs17j120 cs17j121 cs17j409 cs17j122 cs17j123.

compute w1_08_teamsport =0.

compute w1_08_individueelvanuitthuis =0.

compute w1_08_individueelnietvanuitthuis =0.

compute w1_08_overigsport =0.

compute w2_09_teamsport =0.

compute w2_09_individueelvanuitthuis =0.

compute w2_09_individueelnietvanuitthuis =0.

compute w2_09_overigsport =0.

compute w3_10_teamsport =0.

compute w3_10_individueelvanuitthuis =0.

compute w3_10_individueelnietvanuitthuis =0.

compute w3_10_overigsport =0.

compute w4_11_teamsport =0.

compute w4_11_individueelvanuitthuis =0.

compute w4_11_individueelnietvanuitthuis =0.

compute w4_11_overigsport =0.

compute w5_12_teamsport =0.

compute w5_12_individueelvanuitthuis =0.

compute w5_12_individueelnietvanuitthuis =0.

compute w5_12_overigsport =0.

compute w6_13_teamsport =0.

compute w6_13_individueelvanuitthuis =0.

compute w6_13_individueelnietvanuitthuis =0.

compute w6_13_overigsport =0.

compute w7_14_teamsport =0.

compute w7_14_individueelvanuitthuis =0.

compute w7_14_individueelnietvanuitthuis =0.

compute w7_14_overigsport =0.

compute w8_15_teamsport =0.

compute w8_15_individueelvanuitthuis =0.

compute w8_15_individueelnietvanuitthuis =0.

compute w8_15_overigsport =0.

compute w9_16_teamsport =0.

compute w9_16_individueelvanuitthuis =0.

compute w9_16_individueelnietvanuitthuis =0.

compute w9_16_overigsport =0.

compute w10_17_teamsport =0.

compute w10_17_individueelvanuitthuis =0.

compute w10_17_individueelnietvanuitthuis =0.

compute w10_17_overigsport =0.

compute w11_18_teamsport =0.

compute w11_18_individueelvanuitthuis =0.

compute w11_18_individueelnietvanuitthuis =0.

compute w11_18_overigsport =0.

compute w12_19_teamsport =0.

compute w12_19_individueelvanuitthuis =0.

compute w12_19_individueelnietvanuitthuis =0.

compute w12_19_overigsport =0.

if (cs08a106=1) or (cs08a107 =1) or (cs08a108 =1) or (cs08a109=1) or (cs08a110=1) or (cs08a113=1) w1_08_teamsport =1.

if (cs08a114=1) or (cs08a115 =1) or (cs08a118 =1) or (cs08a119 =1) w1_08_individueelvanuitthuis =1.

if (cs08a111 =1) or (cs08a112=1) or (cs08a116=1) or (cs08a117 =1) or (cs08a120 =1) or (cs08a121 =1) w1_08_individueelnietvanuitthuis =1.

if (cs08a122 =1) w1_08_overigsport =1.

if (cs09b106=1) or (cs09b107 =1) or (cs09b108 =1) or (cs09b109=1) or (cs09b110=1) or (cs09b113=1) w2_09_teamsport =1.

if (cs09b114=1) or (cs09b115 =1) or (cs09b118 =1) or (cs09b119 =1) w2_09_individueelvanuitthuis =1.

if (cs09b111 =1) or (cs09b112=1) or (cs09b116=1) or (cs09b117 =1) or (cs09b120 =1) or (cs09b121 =1) w2_09_individueelnietvanuitthuis =1.

if (cs09b122 =1) w2_09_overigsport =1.

if (cs10c106=1) or (cs10c107 =1) or (cs10c108 =1) or (cs10c109=1) or (cs10c110=1) or (cs10c113=1) w3_10_teamsport =1.

if (cs10c114=1) or (cs10c115 =1) or (cs10c118 =1) or (cs10c119 =1) w3_10_individueelvanuitthuis =1.

if (cs10c111 =1) or (cs10c112=1) or (cs10c116=1) or (cs10c117 =1) or (cs10c120 =1) or (cs10c121 =1) w3_10_individueelnietvanuitthuis =1.

if (cs10c122 =1) w3_10_overigsport =1.

if (cs11d106=1) or (cs11d107 =1) or (cs11d108 =1) or (cs11d109=1) or (cs11d110=1) or (cs11d113=1) w4_11_teamsport =1.

if (cs11d114=1) or (cs11d115 =1) or (cs11d118 =1) or (cs11d119 =1) w4_11_individueelvanuitthuis =1.

if (cs11d111 =1) or (cs11d112=1) or (cs11d116=1) or (cs11d117 =1) or (cs11d120 =1) or (cs11d121 =1) or (cs11d409 =1) w4_11_individueelnietvanuitthuis =1.

if (cs11d122 =1) w4_11_overigsport =1.

if (cs12e106=1) or (cs12e107 =1) or (cs12e108 =1) or (cs12e109=1) or (cs12e110=1) or (cs12e113=1) w5_12_teamsport =1.

if (cs12e114=1) or (cs12e115 =1) or (cs12e118 =1) or (cs12e119 =1) w5_12_individueelvanuitthuis =1.

if (cs12e111 =1) or (cs12e112=1) or (cs12e116=1) or (cs12e117 =1) or (cs12e120 =1) or (cs12e121 =1) or (cs12e409 =1) w5_12_individueelnietvanuitthuis =1.

if (cs12e122 =1) w5_12_overigsport =1.

if (cs13f106=1) or (cs13f107 =1) or (cs13f108 =1) or (cs13f109=1) or (cs13f110=1) or (cs13f113=1) w6_13_teamsport =1.

if (cs13f114=1) or (cs13f115 =1) or (cs13f118 =1) or (cs13f119 =1) w6_13_individueelvanuitthuis =1.

if (cs13f111 =1) or (cs13f112=1) or (cs13f116=1) or (cs13f117 =1) or (cs13f120 =1) or (cs13f121 =1) or (cs13f409 =1) w6_13_individueelnietvanuitthuis =1.

if (cs13f122 =1) w6_13_overigsport =1.

if (cs14g106=1) or (cs14g107 =1) or (cs14g108 =1) or (cs14g109=1) or (cs14g110=1) or (cs14g113=1) w7_14_teamsport =1.

if (cs14g114=1) or (cs14g115 =1) or (cs14g118 =1) or (cs14g119 =1) w7_14_individueelvanuitthuis =1.

if (cs14g111 =1) or (cs14g112=1) or (cs14g116=1) or (cs14g117 =1) or (cs14g120 =1) or (cs14g121 =1) or (cs14g409 =1) w7_14_individueelnietvanuitthuis =1.

if (cs14g122 =1) w7_14_overigsport =1.

if (cs15h106=1) or (cs15h107 =1) or (cs15h108 =1) or (cs15h109=1) or (cs15h110=1) or (cs15h113=1) w8_15_teamsport =1.

if (cs15h114=1) or (cs15h115 =1) or (cs15h118 =1) or (cs15h119 =1) w8_15_individueelvanuitthuis =1.

if (cs15h111 =1) or (cs15h112=1) or (cs15h116=1) or (cs15h117 =1) or (cs15h120 =1) or (cs15h121 =1) or (cs15h409 =1) w8_15_individueelnietvanuitthuis =1.

if (cs15h122 =1) w8_15_overigsport =1.

if (cs16i106=1) or (cs16i107 =1) or (cs16i108 =1) or (cs16i109=1) or (cs16i110=1) or (cs16i113=1) w9_16_teamsport =1.

if (cs16i114=1) or (cs16i115 =1) or (cs16i118 =1) or (cs16i119 =1) w9_16_individueelvanuitthuis =1.

if (cs16i111 =1) or (cs16i112=1) or (cs16i116=1) or (cs16i117 =1) or (cs16i120 =1) or (cs16i121 =1) or (cs16i409 =1) w9_16_individueelnietvanuitthuis =1.

if (cs16i122 =1) w9_16_overigsport =1.

if (cs17j106=1) or (cs17j107 =1) or (cs17j108 =1) or (cs17j109=1) or (cs17j110=1) or (cs17j113=1) w10_17_teamsport =1.

if (cs17j114=1) or (cs17j115 =1) or (cs17j118 =1) or (cs17j119 =1) w10_17_individueelvanuitthuis =1.

if (cs17j111 =1) or (cs17j112=1) or (cs17j116=1) or (cs17j117 =1) or (cs17j120 =1) or (cs17j121 =1) or (cs17j409 =1) w10_17_individueelnietvanuitthuis =1.

if (cs17j122 =1) w10_17_overigsport =1.

if (cs18k106=1) or (cs18k107 =1) or (cs18k108 =1) or (cs18k109=1) or (cs18k110=1) or (cs18k113=1) w11_18_teamsport =1.

if (cs18k114=1) or (cs18k115 =1) or (cs18k118 =1) or (cs18k119 =1) w11_18_individueelvanuitthuis =1.

if (cs18k111 =1) or (cs18k112=1) or (cs18k116=1) or (cs18k117 =1) or (cs18k120 =1) or (cs18k121 =1) or (cs18k409 =1) w11_18_individueelnietvanuitthuis =1.

if (cs18k122 =1) w11_18_overigsport =1.

if (cs19l106=1) or (cs19l107 =1) or (cs19l108 =1) or (cs19l109=1) or (cs19l110=1) or (cs19l113=1) w12_19_teamsport =1.

if (cs19l114=1) or (cs19l115 =1) or (cs19l118 =1) or (cs19l119 =1) w12_19_individueelvanuitthuis =1.

if (cs19l111 =1) or (cs19l112=1) or (cs19l116=1) or (cs19l117 =1) or (cs19l120 =1) or (cs19l121 =1) or (cs19l409 =1) w12_19_individueelnietvanuitthuis =1.

if (cs19l122 =1) w12_19_overigsport =1.

des w1_08_teamsport to w12_19_overigsport.

*des cs08a104 cs08a105 cs09b104 cs09b105 cs10c104 cs10c105 cs11d104 cs11d105 cs12e104 cs12e105 cs13f104 cs13f105 cs14g104 cs14g105 cs15h104 cs15h105 cs16i104 cs16i105 cs17j104 cs17j105.

*hebben partner & samenwonen partner cf08a024 cf08a025 cf09b024 cf09b025 cf10c024 cf10c025 cf11d024 cf11d025 cf12e024 cf12e025 cf13f024 cf13f025 cf14g024 cf14g025 cf15h024 cf15h025 cf16i024 cf16i025 cf17j024 cf17j025.

*geb jaar 1e kind cf08a037 cf09b037 cf10c037 cf11d037 cf12e037 cf13f037 cf14g037 cf15h456 cf16i456 cf17j456.

*fre cs08a104 cs08a105 cs09b104 cs09b105 cs10c104 cs10c105 cs11d104 cs11d105 cs12e104 cs12e105 cs13f104 cs13f105 cs14g104 cs14g105 cs15h104 cs15h105 cs16i104 cs16i105 cs17j104 cs17j105.

*werkuren .

*fre cw08a126 cw08a127 cw09b126 cw09b127 cw10c126 cw10c127 cw11d126 cw11d127 cw12e126 cw12e127 cw13f126 cw13f127 cw14g126 cw14g127 cw15h126 cw15h127 cw16i126 cw16i127 cw17j126 cw17j127 cw18k126 cw18k127 cw19l126 cw19l127.

*fre cw08a005 cw09b005 cw10c005 cw11d005 cw12e005 cw13f005 cw14g005 cw15h005 cw16i005 cw17j005 cw18k005 cw19l008.

SAVE OUTFILE='C:\Users\U046129\Dropbox\NWA TRAIL WP3\LISS DATA\LISS w1 w12 beknopt.sav'

/keep= nomem_encr female checkgebjaar cf08a024 cf08a025 cf09b024 cf09b025 cf10c024 cf10c025 cf11d024 cf11d025 cf12e024 cf12e025 cf13f024 cf13f025 cf14g024 cf14g025 cf15h024 cf15h025 cf16i024

cf16i025 cf17j024 cf17j025 cf18k024 cf18k025 cf19l024 cf19l025

cs08a104 cs08a105 cs09b104 cs09b105 cs10c104 cs10c105 cs11d104 cs11d105 cs12e104 cs12e105 cs13f104 cs13f105 cs14g104 cs14g105 cs15h104 cs15h105 cs16i104 cs16i105 cs17j104 cs17j105 cs18k104 cs18k105 cs19l104 cs19l105

cw08a126 cw08a127 cw09b126 cw09b127 cw10c126 cw10c127 cw11d126 cw11d127 cw12e126 cw12e127 cw13f126 cw13f127 cw14g126 cw14g127 cw15h126 cw15h127 cw16i126 cw16i127 cw17j126 cw17j127 cw18k126 cw18k127 cw19l126 cw19l127

cw08a005 cw09b005 cw10c005 cw11d005 cw12e005 cw13f005 cw14g005 cw15h005 cw16i005 cw17j005 cw18k005 cw19l008

w1_08_teamsport w1_08_individueelvanuitthuis w1_08_individueelnietvanuitthuis w1_08_overigsport

w2_09_teamsport w2_09_individueelvanuitthuis w2_09_individueelnietvanuitthuis w2_09_overigsport

w3_10_teamsport w3_10_individueelvanuitthuis w3_10_individueelnietvanuitthuis w3_10_overigsport

w4_11_teamsport w4_11_individueelvanuitthuis w4_11_individueelnietvanuitthuis w4_11_overigsport

w5_12_teamsport w5_12_individueelvanuitthuis w5_12_individueelnietvanuitthuis w5_12_overigsport

w6_13_teamsport w6_13_individueelvanuitthuis w6_13_individueelnietvanuitthuis w6_13_overigsport

w7_14_teamsport w7_14_individueelvanuitthuis w7_14_individueelnietvanuitthuis w7_14_overigsport

w8_15_teamsport w8_15_individueelvanuitthuis w8_15_individueelnietvanuitthuis w8_15_overigsport

w9_16_teamsport w9_16_individueelvanuitthuis w9_16_individueelnietvanuitthuis w9_16_overigsport

w10_17_teamsport w10_17_individueelvanuitthuis w10_17_individueelnietvanuitthuis w10_17_overigsport

w11_18_teamsport w11_18_individueelvanuitthuis w11_18_individueelnietvanuitthuis w11_18_overigsport

w12_19_teamsport w12_19_individueelvanuitthuis w12_19_individueelnietvanuitthuis w12_19_overigsport

burgstat checkkind1 checkkind2 checkkind3 checkkind4 checkkind5 belbezig oplcat oplzon nettohh_f

/COMPRESSED.

get file ='C:\Users\U046129\Dropbox\NWA TRAIL WP3\LISS DATA\LISS w1 w12 beknopt.sav'.

exe.

rename variables (nomem_encr = id).

rename variables (checkgebjaar = gebjaar).

rename variables (cf08a024 = partner_2008).

rename variables (cf08a025 = samenwonen_2008).

rename variables (cf09b024 = partner_2009).

rename variables (cf09b025 = samenwonen_2009).

rename variables (cf10c024 = partner_2010).

rename variables (cf10c025 = samenwonen_2010).

rename variables (cf11d024 = partner_2011) .

rename variables (cf11d025 = samenwonen_2011).

rename variables (cf12e024 = partner_2012).

rename variables (cf12e025 = samenwonen_2012).

rename variables (cf13f024 = partner_2013).

rename variables (cf13f025= samenwonen_2013).

rename variables (cf14g024= partner_2014).

rename variables (cf14g025=samenwonen_2014).

rename variables (cf15h024= partner_2015).

rename variables (cf15h025 = samenwonen_2015).

rename variables (cf16i024=partner_2016).

rename variables (cf16i025=samenwonen_2016).

rename variables (cf17j024=partner_2017).

rename variables (cf17j025=samenwonen_2017).

rename variables (cf18k024=partner_2018).

rename variables (cf18k025=samenwonen_2018) (cf19l024=partner_2019) (cf19l025=samenwonen_2019) (cs08a104 =sporten_2008) (cs08a105=urensport_2008) (cs09b104 =sporten_2009)

(cs09b105 = urensport_2009) (cs10c104 = sporten_2010) (cs10c105= urensport_2010) (cs11d104= sporten_2011) (cs11d105=urensport_2011) (cs12e104=sporten_2012) (cs12e105 = urensport_2012)

(cs13f104 = sporten_2013) (cs13f105 = urensport_2013) (cs14g104 = sporten_2014) (cs14g105 = urensport_2014) (cs15h104 = sporten_2015) (cs15h105 =urensport_2015) (cs16i104 = sporten_2016) (cs16i105 = urensport_2016)

(cs17j104 = sporten_2017) (cs17j105 = urensport_2017) (cs18k104 = sporten_2018) (cs18k105 = urensport_2018) (cs19l104 = sporten_2019) (cs19l105 = urensport_2019).

rename variables (w1_08_teamsport = teamsport_2008) (w1_08_individueelvanuitthuis =individueelvanuitthuis_2008)

(w1_08_individueelnietvanuitthuis =individueelnietvanuitthuis_2008)

(w1_08_overigsport = overigsport_2008) (w2_09_teamsport = teamsport_2009) (w2_09_individueelvanuitthuis = individueelvanuitthuis_2009) (w2_09_individueelnietvanuitthuis = individueelnietvanuitthuis_2009) (w2_09_overigsport = overigsport_2009)

(w3_10_teamsport = teamsport_2010) (w3_10_individueelvanuitthuis = individueelvanuitthuis_2010) (w3_10_individueelnietvanuitthuis = individueelnietvanuitthuis_2010) (w3_10_overigsport = overigsport_2010)

(w4_11_teamsport = teamsport_2011) (w4_11_individueelvanuitthuis = individueelvanuitthuis_2011) (w4_11_individueelnietvanuitthuis = individueelnietvanuitthuis_2011) (w4_11_overigsport = overigsport_2011)

(w5_12_teamsport = teamsport_2012) (w5_12_individueelvanuitthuis = individueelvanuitthuis_2012) (w5_12_individueelnietvanuitthuis = individueelnietvanuitthuis_2012) (w5_12_overigsport = overigsport_2012)

(w6_13_teamsport = teamsport_2013) (w6_13_individueelvanuitthuis = individueelvanuitthuis_2013) (w6_13_individueelnietvanuitthuis = individueelnietvanuitthuis_2013) (w6_13_overigsport = overigsport_2013)

(w7_14_teamsport = teamsport_2014) (w7_14_individueelvanuitthuis = individueelvanuitthuis_2014) (w7_14_individueelnietvanuitthuis = individueelnietvanuitthuis_2014) (w7_14_overigsport = overigsport_2014)

(w8_15_teamsport = teamsport_2015) (w8_15_individueelvanuitthuis = individueelvanuitthuis_2015) (w8_15_individueelnietvanuitthuis = individueelnietvanuitthuis_2015) (w8_15_overigsport = overigsport_2015)

(w9_16_teamsport = teamsport_2016) (w9_16_individueelvanuitthuis = individueelvanuitthuis_2016) (w9_16_individueelnietvanuitthuis = individueelnietvanuitthuis_2016) (w9_16_overigsport = overigsport_2016)

(w10_17_teamsport = teamsport_2017) (w10_17_individueelvanuitthuis = individueelvanuitthuis_2017) (w10_17_individueelnietvanuitthuis = individueelnietvanuitthuis_2017) (w10_17_overigsport = overigsport_2017).

rename variables (w11_18_teamsport = teamsport_2018) (w11_18_individueelvanuitthuis = individueelvanuitthuis_2018) (w11_18_individueelnietvanuitthuis = individueelnietvanuitthuis_2018) (w11_18_overigsport = overigsport_2018)

(w12_19_teamsport = teamsport_2019) (w12_19_individueelvanuitthuis = individueelvanuitthuis_2019) (w12_19_individueelnietvanuitthuis = individueelnietvanuitthuis_2019) (w12_19_overigsport = overigsport_2019)

(cw08a126 = urencontract_2008) (cw08a127 = urenwerk_2008) (cw09b126 = urencontract_2009) (cw09b127 = urenwerk_2009) (cw10c126 = urencontract_2010) (cw10c127 = urenwerk_2010)

(cw11d126 = urencontract_2011) (cw11d127 = urenwerk_2011) (cw12e126 = urencontract_2012) (cw12e127 = urenwerk_2012) (cw13f126 = urencontract_2013) (cw13f127 = urenwerk_2013)

(cw14g126 = urencontract_2014) (cw14g127 = urenwerk_2014) (cw15h126 = urencontract_2015) (cw15h127 = urenwerk_2015) (cw16i126 = urencontract_2016) (cw16i127 = urenwerk_2016)

(cw17j126 = urencontract_2017) (cw17j127 = urenwerk_2017) (cw18k126 = urencontract_2018) (cw18k127 = urenwerk_2018) (cw19l126 = urencontract_2019) (cw19l127 = urenwerk_2019) .

rename variables (cw08a005 = opl_2008) (cw09b005= opl_2009) (cw10c005 = opl_2010) (cw11d005 = opl_2011) (cw12e005 = opl_2012) (cw13f005 = opl_2013) (cw14g005 = opl_2014)

(cw15h005 = opl_2015) (cw16i005 = opl_2016) (cw17j005 = opl_2017) (cw18k005 = opl_2018) (cw19l008 = opl_2019).

EXE.

recode urensport_2008 urensport_2009 urensport_2010 urensport_2011 urensport_2012 urensport_2013 urensport_2014 urensport_2015 urensport_2016 urensport_2017 urensport_2018 urensport_2019 (25 thru highest = sysmis) (else = copy) into

urensport2_2008 urensport2_2009 urensport2_2010 urensport2_2011 urensport2_2012 urensport2_2013 urensport2_2014 urensport2_2015 urensport2_2016 urensport2_2017 urensport2_2018 urensport2_2019.

if (sporten_2008) = 2 urensport2_2008 =0.

if (sporten_2009) = 2 urensport2_2009 =0.

if (sporten_2010) = 2 urensport2_2010 =0.

if (sporten_2011) = 2 urensport2_2011 =0.

if (sporten_2012) = 2 urensport2_2012 =0.

if (sporten_2013) = 2 urensport2_2013 =0.

if (sporten_2014) = 2 urensport2_2014 =0.

if (sporten_2015) = 2 urensport2_2015 =0.

if (sporten_2016) = 2 urensport2_2016 =0.

if (sporten_2017) = 2 urensport2_2017 =0.

if (sporten_2018) = 2 urensport2_2018 =0.

if (sporten_2019) = 2 urensport2_2019 =0.

*fre urensport2_2008 to urensport2_2019.

compute verschilsporten_2008 = 0.

compute verschilsporten_2009 = urensport2_2008 - urensport2_2009.

compute verschilsporten_2010 = urensport2_2009 - urensport2_2010.

compute verschilsporten_2011 = urensport2_2010 - urensport2_2011.

compute verschilsporten_2012 = urensport2_2011 - urensport2_2012.

compute verschilsporten_2013 = urensport2_2012 - urensport2_2013.

compute verschilsporten_2014 = urensport2_2013 - urensport2_2014.

compute verschilsporten_2015 = urensport2_2014 - urensport2_2015.

compute verschilsporten_2016 = urensport2_2015 - urensport2_2016.

compute verschilsporten_2017 = urensport2_2016 - urensport2_2017.

compute verschilsporten_2018 = urensport2_2017 - urensport2_2018.

compute verschilsporten_2019 = urensport2_2018 - urensport2_2019.

*des verschilsporten_2009 to verschilsporten_2019.

VARSTOCASES

/make partner from partner_2008 partner_2009 partner_2010 partner_2011 partner_2012 partner_2013 partner_2014 partner_2015 partner_2016 partner_2017 partner_2018 partner_2019

/make samenwonen from samenwonen_2008 samenwonen_2009 samenwonen_2010 samenwonen_2011 samenwonen_2012 samenwonen_2013 samenwonen_2014 samenwonen_2015 samenwonen_2016 samenwonen_2017 samenwonen_2018 samenwonen_2019

/make sporten from sporten_2008 sporten_2009 sporten_2010 sporten_2011 sporten_2012 sporten_2013 sporten_2014 sporten_2015 sporten_2016 sporten_2017 sporten_2018 sporten_2019

/make urensport from urensport_2008 urensport_2009 urensport_2010 urensport_2011 urensport_2012 urensport_2013 urensport_2014 urensport_2015 urensport_2016 urensport_2017 urensport_2018 urensport_2019

/make urensport2 from urensport2_2008 urensport2_2009 urensport2_2010 urensport2_2011 urensport2_2012 urensport2_2013 urensport2_2014 urensport2_2015 urensport2_2016 urensport2_2017 urensport2_2018 urensport2_2019

/make verschilsporten from verschilsporten_2008 verschilsporten_2009 verschilsporten_2010 verschilsporten_2011 verschilsporten_2012 verschilsporten_2013 verschilsporten_2014 verschilsporten_2015 verschilsporten_2016 verschilsporten_2017

verschilsporten_2018 verschilsporten_2019

/make teamsport from teamsport_2008 teamsport_2009 teamsport_2010 teamsport_2011 teamsport_2012 teamsport_2013 teamsport_2014 teamsport_2015 teamsport_2016 teamsport_2017 teamsport_2018 teamsport_2019

/make individueelvanuitthuis from individueelvanuitthuis_2008 individueelvanuitthuis_2009 individueelvanuitthuis_2010 individueelvanuitthuis_2011 individueelvanuitthuis_2012 individueelvanuitthuis_2013

individueelvanuitthuis_2014 individueelvanuitthuis_2015 individueelvanuitthuis_2016 individueelvanuitthuis_2017 individueelvanuitthuis_2018 individueelvanuitthuis_2019

/make individueelnietvanuitthuis from individueelnietvanuitthuis_2008 individueelnietvanuitthuis_2009 individueelnietvanuitthuis_2010 individueelnietvanuitthuis_2011 individueelnietvanuitthuis_2012 individueelnietvanuitthuis_2013

individueelnietvanuitthuis_2014 individueelnietvanuitthuis_2015 individueelnietvanuitthuis_2016 individueelnietvanuitthuis_2017 individueelnietvanuitthuis_2018 individueelnietvanuitthuis_2019

/make overigsport from overigsport_2008 overigsport_2009 overigsport_2010 overigsport_2011 overigsport_2012 overigsport_2013 overigsport_2014 overigsport_2015 overigsport_2016 overigsport_2017 overigsport_2018 overigsport_2019

/make urencontract from urencontract_2008 urencontract_2009 urencontract_2010 urencontract_2011 urencontract_2012 urencontract_2013 urencontract_2014 urencontract_2015 urencontract_2016 urencontract_2017 urencontract_2018 urencontract_2019

/make urenwerk from urenwerk_2008 urenwerk_2009 urenwerk_2010 urenwerk_2011 urenwerk_2012 urenwerk_2013 urenwerk_2014 urenwerk_2015 urenwerk_2016 urenwerk_2017 urenwerk_2018 urenwerk_2019

/make opleiding from opl_2008 opl_2009 opl_2010 opl_2011 opl_2012 opl_2013 opl_2014 opl_2015 opl_2016 opl_2017 opl_2018 opl_2019

/index= jaar(12).

exe.

recode jaar (1=2008) (2=2009) (3=2010) (4=2011) (5=2012) (6=2013) (7=2014) (8=2015) (9=2016) (10=2017) (11=2018) (12=2019).

exe.

des checkkind1 to checkkind5.

compute volgok = (checkkind2-checkkind1).

fre volgok.

temp.

select if (volgok<0).

fre id.

use all.

do if (id = 801589) or (id = 829888) or (id = 829943) or (id = 849230) or (id = 861566) or (id = 877017) or (id = 888793) or (id = 888839).

compute checkkindv1 = checkkind2.

compute checkkindv2 = checkkind1.

compute checkkind1 = checkkindv1.

compute checkkind2 = checkkindv2.

end if.

compute volgok2 = (checkkind2-checkkind1).

fre volgok2.

compute bc1 = 0.

if (checkkind1 = jaar) bc1 = 1.

*fre bc1.

compute bc2 = 0.

if (checkkind2 = jaar) bc2 = 1.

*fre bc2.

compute bc3 = 0.

if (checkkind3 = jaar) bc3 = 1.

*fre bc3.

compute bc4 = 0.

if (checkkind4 = jaar) bc4 = 1.

*fre bc4.

compute bc5 = 0.

if (checkkind5 = jaar) bc5 = 1.

*fre bc5.

compute bc =0.

if (checkkind1 = jaar) or (checkkind2 = jaar) or (checkkind3 = jaar) or (checkkind4 = jaar) or (checkkind5 = jaar) bc=1.

*fre bc.

compute yab1 = jaar - checkkind1.

recode yab1 (lowest thru -1 = sysmis) (else = copy).

*fre yab1.

compute yab2 = jaar - checkkind2.

recode yab2 (lowest thru -1 = sysmis) (else = copy).

*fre yab2.

compute yab3 = jaar - checkkind3.

recode yab3 (lowest thru -1 = sysmis) (else = copy).

*fre yab3.

compute yab4 = jaar - checkkind4.

recode yab4 (lowest thru -1 = sysmis) (else = copy).

*fre yab4.

compute yab5 = jaar - checkkind5.

recode yab5 (lowest thru -1 = sysmis) (else = copy).

*fre yab5.

compute yab = yab1.

if (yab2<yab1) yab = yab2.

if (yab3<yab2) yab = yab3.

if (yab4<yab3) yab = yab4.

if (yab5<yab4) yab = yab5.

fre yab.

SAVE TRANSLATE OUTFILE='C:\Users\U046129\Dropbox\NWA TRAIL WP3\LISS DATA\Liss 1 to 12 long 20210712.dta'

/TYPE=STATA

/VERSION=14

/EDITION=SE

/MAP

/REPLACE

/DROP=volgok checkkindv1 checkkindv2 volgok2.

*****************************.

*Analyses in Stata

******************************

use "C:\Users\U046129\Dropbox\NWA TRAIL WP3\LISS DATA\Liss 1 to 12 long 20210712.dta", clear

sort id jaar

xtset id jaar

gen sporten2 =.

replace sporten2 = 1 if sporten ==1

replace sporten2 = 0 if sporten ==2

gen bavmbo = .

replace bavmbo = 1 if oplzon == 1 | oplzon == 2

replace bavmbo = 0 if oplzon == 3 | oplzon == 4 | oplzon == 5 | oplzon == 6 | oplzon == 7 | oplzon == 8 | oplzon == 9

gen hvmbo = .

replace hvmbo = 1 if oplzon ==3 | oplzon ==4

replace hvmbo = 0 if oplzon ==1 | oplzon ==2| oplzon ==5 | oplzon ==6| oplzon ==7| oplzon ==8| oplzon ==9

gen hbowo =.

replace hbowo = 1 if oplzon == 5 | oplzon ==6

replace hbowo = 0 if oplzon == 1 | oplzon == 2 | oplzon == 3| oplzon == 4| oplzon == 7| oplzon == 8| oplzon == 9

gen opljaar = .

replace opljaar = 4 if oplcat ==1

replace opljaar = 6 if oplcat ==2

replace opljaar = 8 if oplcat ==3

replace opljaar = 10.5 if oplcat ==4

replace opljaar = 15 if oplcat ==5

replace opljaar = 16.5 if oplcat ==6

gen opljaar2 = .

replace opljaar2 = 0 if opleiding == 1

replace opljaar2 = 8 if opleiding == 2

replace opljaar2 = 12 if opleiding == 3

replace opljaar2 = 14 if opleiding == 4

replace opljaar2 = 15 if opleiding == 5

replace opljaar2 = 16 if opleiding == 6

replace opljaar2 = 16 if opleiding == 7

replace opljaar2 = 16 if opleiding == 8

replace opljaar2 = 16 if opleiding == 9

replace opljaar2 = 16 if opleiding == 10

replace opljaar2 = 17 if opleiding == 11

replace opljaar2 = 17 if opleiding == 12

replace opljaar2 = 18 if opleiding == 13

replace opljaar2 = 18 if opleiding == 14

replace opljaar2 = 19 if opleiding == 15

replace opljaar2 = 20 if opleiding == 16

replace opljaar2 = 20 if opleiding == 17

replace opljaar2 = 21 if opleiding == 18

replace opljaar2 = 21 if opleiding == 19

replace opljaar2 = 21 if opleiding == 20

replace opljaar2 = 21 if opleiding == 21

replace opljaar2 = 23 if opleiding == 22

replace opljaar2 = 23 if opleiding == 23

replace opljaar2 = 24 if opleiding == 24

replace opljaar2 = 24 if opleiding == 25

replace opljaar2 = 28 if opleiding == 26

replace opljaar2 = . if opleiding == 27

replace opljaar2 = . if opleiding == 28

gen leeftijd = jaar - gebjaar

gen onder25 = .

replace onder25 = 1 if leeftijd <26

gen lft2635 = .

replace lft2635 = 1 if leeftijd >=26 & leeftijd <36

replace lft2635 = 0 if leeftijd <26

replace lft2635 = 0 if leeftijd >=36

gen lft3645 = .

replace lft3645 = 1 if leeftijd >=36 & leeftijd <46

replace lft3645 = 0 if leeftijd <36

replace lft3645 = 0 if leeftijd >=46

gen lft4655 = .

replace lft4655 = 1 if leeftijd >=46 & leeftijd <56

replace lft4655 = 0 if leeftijd <46

replace lft4655 = 0 if leeftijd >=56

gen boven55 = .

replace boven55 = 1 if leeftijd >= 56

replace boven55 = 0 if leeftijd <56

gen werk =.

replace werk = 1 if belbezig ==1

replace werk = 0 if belbezig >1 & belbezig <14

gen sportencat =.

replace sportencat = 0 if urensport2 ==0

replace sportencat = 1 if urensport2 >0 & urensport2 <2

replace sportencat = 2 if urensport2 >=2 & urensport2 <4

replace sportencat = 3 if urensport2 >=4 & urensport2 <25

gen sportencat2 =.

replace sportencat2 = 0 if urensport2 ==0

replace sportencat2 = 1 if urensport2 >0 & urensport2 <2

replace sportencat2 = 2 if urensport2 >=2 & urensport2 <4

replace sportencat2 = 3 if urensport2 >=4 & urensport2 <8.5

replace sportencat2 = 4 if urensport2 >=8.5 & urensport2 <25

gen sportencat3 =.

replace sportencat3 = 0 if urensport2 ==0

replace sportencat3 = 1 if urensport2 >0 & urensport2 <2

replace sportencat3 = 2 if urensport2 >=2 & urensport2 <5

replace sportencat3 = 3 if urensport2 >=5 & urensport2 <10

replace sportencat3 = 4 if urensport2 >=10 & urensport2 <25

gen uren = .

replace uren = 0 if werk == 0

replace uren = urenwerk if werk ==1

replace uren = 10 if urenwerk == 100

replace uren = 15 if urenwerk ==150

drop if urensport2 ==.

drop if partner ==.

drop if werk ==.

drop if opljaar == .

drop if uren == .

drop if bc == .

drop if opljaar2 ==.

** interacties

gen bcopljaar = bc * opljaar2

gen bcfemale = bc * female

gen bc1opljaar = bc1 * opljaar2

gen bc1female = bc1 * female

gen bc2opljaar = bc2 * opljaar2

gen bc2female = bc2 * female

gen yabfemale = yab * female

gen yabopljaar = yab *opljaar2

gen yab1female = yab1 * female

gen yab1opljaar = yab1 *opljaar2

gen yab2female2 = yab * female

gen yab2opljaar2 = yab *opljaar2

bysort id jaar : gen validobservation = sum(sporten == 1)

bysort id : egen valid = sum( validobservation )

** interactie term ooit ouder * jaren na geboorte. Jaren na geboorte wat nu officieel 0 is 1 maken zodat ouders dan een 1 krijgen

*verschil score om een effect van opleiding te zien i.v.m. plafond effect hoger opgeleiden?

drop if valid <2

**leeftijd bij yab opnemen als categories ivm samenhang tussen leeftijd en jaren na geboorte

*gen bchvmbo = bc * hvmbo

*gen bchbowo = bc * hbowo

*gen bcbavmbo = bc * bavmbo

*gen yabhvmbo = yab * hvmbo

*gen yabhbowo = yab * hbowo

*gen yabbavmbo = yab * bavmbo

*al dan niet sporten op basis van vraag

xtlogit sporten2 bc female leeftijd opljaar2 partner uren if valid >1, vce(r) level(95) or

xtlogit sporten2 bc female bcfemale leeftijd opljaar2 partner uren if valid >1, vce(r) level(95) or

xtlogit sporten2 bc female leeftijd opljaar2 partner uren if valid >1, vce(r) level(95)

xtlogit sporten2 bc female bcfemale leeftijd opljaar2 partner uren if valid >1, vce(r) level(95)

xtlogit sporten2 yab female leeftijd opljaar2 partner uren if valid >1, vce(r) level(95) or

xtlogit sporten2 yab female yabfemale leeftijd opljaar2 partner uren if valid >1, vce(r) level(95) or

xtlogit sporten2 yab female leeftijd opljaar2 partner uren if valid >1, vce(r)

xtlogit sporten2 yab female yabfemale leeftijd opljaar2 partner uren if valid >1, vce(r)

*urensport in categorieen

xtologit sportencat bc female leeftijd opljaar2 partner uren if valid >1, vce(r) level(95) or

xtologit sportencat bc female bcfemale leeftijd opljaar2 partner uren if valid >1, vce(r) level(95) or

xtologit sportencat bc female leeftijd opljaar2 partner uren if valid >1, vce(r)

xtologit sportencat bc female bcfemale leeftijd opljaar2 partner uren if valid >1, vce(r)

xtologit sportencat yab female leeftijd opljaar2 partner uren if valid >1, vce(r) level(95) or

xtologit sportencat yab female bcfemale leeftijd opljaar2 partner uren if valid >1, vce(r) level(95) or

xtologit sportencat yab female leeftijd opljaar2 partner uren if valid >1, vce(r)

xtologit sportencat yab female bcfemale leeftijd opljaar2 partner uren if valid >1, vce(r)

*sportvorm los van elkaar

xtlogit teamsport bc female leeftijd opljaar2 partner uren if valid >1, vce(r) level(95) or

xtlogit individueelnietvanuitthuis bc female leeftijd opljaar2 partner uren if valid >1, vce(r) level(95) or

xtlogit individueelvanuitthuis bc female leeftijd opljaar2 partner uren if valid >1, vce(r) level(95) or

xtlogit teamsport bc female leeftijd opljaar2 partner uren if valid >1, vce(r)

xtlogit individueelnietvanuitthuis bc female leeftijd opljaar2 partner uren if valid >1, vce(r)

xtlogit individueelvanuitthuis bc female leeftijd opljaar2 partner uren if valid >1, vce(r)

xtlogit teamsport yab female leeftijd opljaar2 partner uren if valid >1, vce(r) level(95) or

xtlogit individueelnietvanuitthuis yab female leeftijd opljaar2 partner uren if valid >1, vce(r) level(95) or

xtlogit individueelvanuitthuis yab female leeftijd opljaar2 partner uren if valid >1, vce(r) level(95) or

xtlogit teamsport yab female leeftijd opljaar2 partner uren if valid >1, vce(r)

xtlogit individueelnietvanuitthuis yab female leeftijd opljaar2 partner uren if valid >1, vce(r)

xtlogit individueelvanuitthuis yab female leeftijd opljaar2 partner uren if valid >1, vce(r)
